# Responses of four dominant dryland plant species to climate change in the Junggar Basin, northwest China

**DOI:** 10.1002/ece3.5817

**Published:** 2019-11-11

**Authors:** Jian Xiao, Anwar Eziz, Heng Zhang, Zhiheng Wang, Zhiyao Tang, Jingyun Fang

**Affiliations:** ^1^ Institute of Ecology College of Urban and Environmental Sciences, and Key Laboratory for Earth Surface Processes of the Ministry of Education Peking University Beijing China

**Keywords:** climate change, distribution, dryland, Junggar Basin, plant species, species distribution model

## Abstract

**Aim:**

Dryland ecosystems are exceedingly sensitive to climate change. Desertification induced by both climate changes and human activities seriously threatens dryland vegetation. However, the impact of climate change on distribution of dryland plant species has not been well documented. Here, we studied the potential distribution of four representative dryland plant species (*Haloxylon ammodendron*, *Anabasis aphylla*, *Calligonum mongolicum*, and *Populus euphratica*) under current and future climate scenarios in a temperate desert region, aiming to improve our understanding of the responses of dryland plant species to climate change and provide guidance for dryland conservation and afforestation.

**Location:**

Junggar Basin, a large desert region in northwestern China.

**Methods:**

Occurrence data of the studied species were collected from an extensive field investigation of 2,516 sampling sites in the Junggar Basin. Ensemble species distribution models using 10 algorithms were developed and used to predict the potential distribution of each studied species under current and future climate scenarios.

**Result:**

*Haloxylon ammodendron* and *A. aphylla* were likely to lose most of their current suitable habitats under future climate scenarios, while *C. mongolicum* and *P. euphratica* were likely to expand their ranges or remain relatively stationary. Variable importance evaluation showed that the most important climate variables influencing species distribution differed across the studied species. These results may be explained by the different ecophysiological characteristics and adaptation strategies to the environment of the four studied species.

**Main conclusions:**

We explored the responses of the representative dryland plant species to climate change in the Junggar Basin in northwestern China. The different changes in suitability of different species imply that policymakers may need to reconsider the selection and combination of the afforestation species used in this area. This study can provide valuable reference for the management and conservation of dryland ecosystems under future climate change scenarios.

## Graphical abstract

Using distribution data of four dominant dryland plant species in a temperate desert region in northwestern China obtained from extensive field survey, we evaluated their responses to future climate change based on ensemble species distribution modeling. We found that these species differed in the direction or extent of habitat change under future climate scenarios.

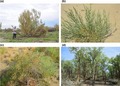

## 1 INTRODUCTION

Drylands cover over 41% of the Earth's land surface (Berdugo, Kefi, Soliveres, & Maestre, 2017) and have been ranked at the forefront of vulnerability to global change (Parry et al., 2007). Desertification induced by both climate changes and human activities has been seriously threatening dryland vegetation (D'Odorico, Bhattachan, Davis, Ravi, & Runyan, 2013). Dryland plant species play a vital role in maintaining ecological stability and preventing natural disasters like sandstorms, heatwaves, and desertification (Sivakumar & Stefanski, 2007). Yet, dryland plant species are subject to strong climatic control and may be more sensitive to climate change compared with species in other ecosystems (Vale & Brito, 2015). However, previous studies on species distribution changes in response to climate change have mainly been focused on ecosystems like temperate forests, boreal forests, and tundra, while the number of studies on drylands is relatively few, and most have been conducted in North American deserts (Lenoir & Svenning, 2015).

In recent years, climate change coupled with human activities has strongly affected the distribution of plant species in the drylands of northwestern China. On the one hand, vegetation coverage reduction and degradation driven by climate change and overexploitation of water resources have been observed (Wang, Pan, Wang, Shen, & Lu, 2013). On the other hand, several drought‐resistant woody plant species have been widely planted since 1970s as large‐scale afforestation efforts to combat desertification in the drylands of northwestern China (Yu & Wang, 2018). Nevertheless, these efforts are not always successful due to a lack of understanding about the habitat suitability of the planted species and their responses to climatic change. High mortality has been reported in numerous cases of afforestation (Cao, 2008). Therefore, more studies are urgently needed to estimate the future dynamics of vegetation and to provide better guidance for ecological conservation and restoration in the drylands of northwestern China.

Junggar Basin, located in northwestern China, contains the second largest desert in China. Compared to the other deserts in Central Asia, the desert of Junggar Basin has extremely diverse plant communities and possesses one of the richest floras in the world's temperate deserts (Zhang & Chen, 2002). Junggar Basin has also developed diverse landscapes and habitats, from Gobi Desert foothills to sandy deserts and oases. Plant communities and environments vary widely between different subregions within the Junggar Basin. Several dominant woody plant species make up the majority of the desert vegetation in the basin (Tang, Yan, & Zhang, 2008). Identifying the suitable habitats of dominant woody plant species and their responses to climate change in the Junggar Basin would provide a useful case to demonstrate the importance of knowledge on species' suitability in restoration in drylands.

**Figure 1:**
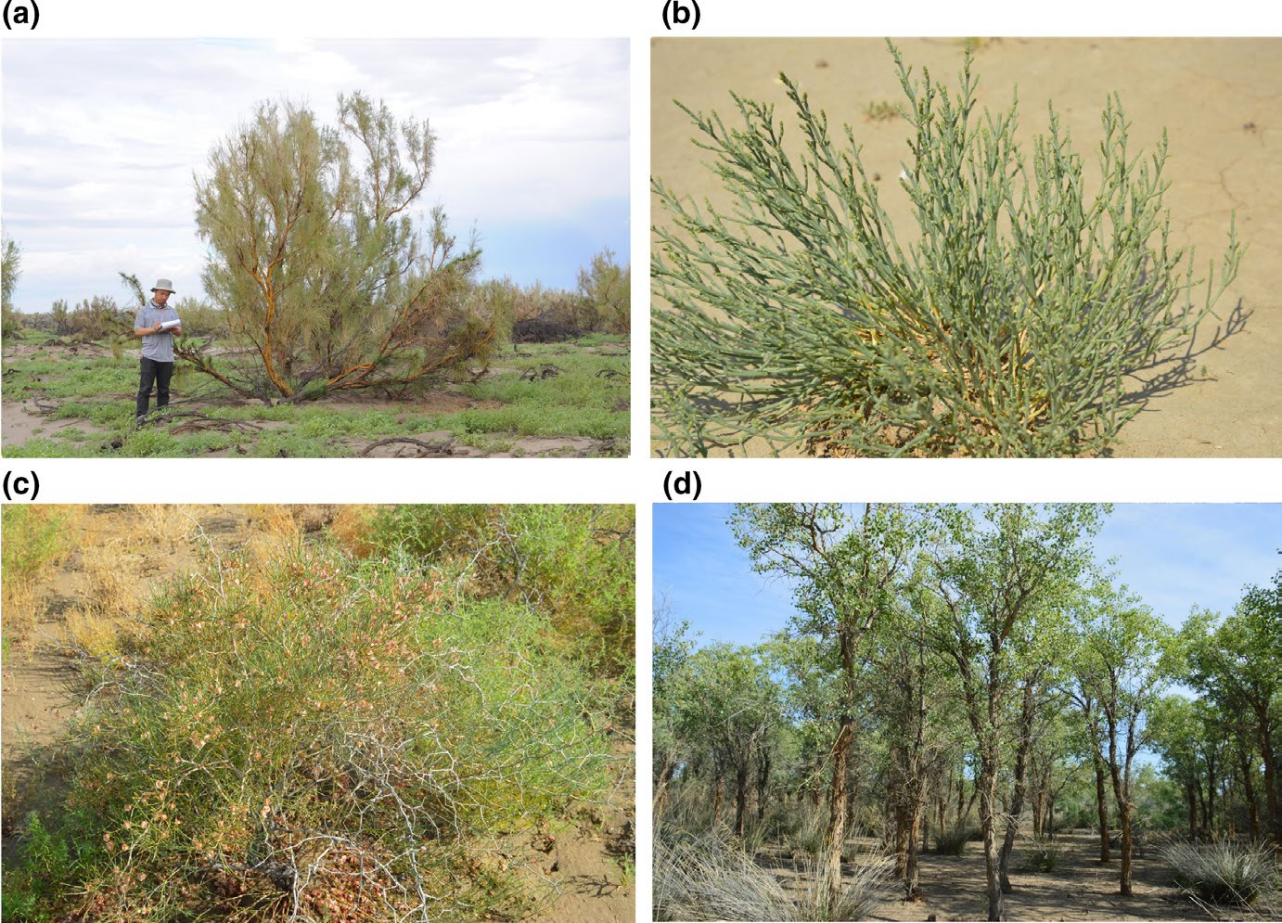
The four studied species: *Haloxylon ammodendron* (a), *Anabasis aphylla* (b), *Calligonum mongolicum* (c), and *Populus euphratica* (d). Photographs taken by Anwar Eziz

Species distribution model (SDM) is based on the concept of ecological niche and can identify the statistical relationships between species occurrence data and environmental variables (Peterson et al., 2011). SDM has been widely used in the prediction of environmental suitability and potential distribution range for species (Dyderski, Paz, Frelich, & Jagodzinski, 2018; Noce, Collalti, & Santini, 2017; Wang, Liu, et al., 2018). In this study, we used comprehensive field investigation data and an ensemble SDM method to predict the potential distribution (i.e., suitable habitat) of four representative woody plant species in the Junggar Basin under the current climate and future climate scenarios. We aim to answer the following questions: (a) How will the dominant plant species of the Junggar Basin respond to climate change? (b) Do these species respond similarly to future climate change? If not, what are the possible reasons? The species studied are *Haloxylon ammodendron*, *Anabasis aphylla*, *Calligonum mongolicum*, and *Populus euphratica* (Figure 1). *H. ammodendron* is a perennial small tree, and *C. mongolicum* is a perennial shrub. Both are dominant species in the southern desert area of the Junggar Basin but with different ecological characteristics (Eziz, 2018). In addition, they are two of the most widely used plantation species in afforestation and ecological restoration in northwestern China (Yu & Wang, 2018). *A. aphylla* is a dominant perennial sub‐shrub species in the Gobi Desert in the northern mountain area of the basin, where the altitude is relatively higher and the climate is colder and wetter than that of the southern desert (Eziz, 2018). *P. euphratica* is the dominant species of the desert riparian forest, which is an important component of arid inland ecosystems (Li, Zhou, Fu, & Chen, 2013).

## 2 METHODS

### 2.1 Study area

**Figure 2:**
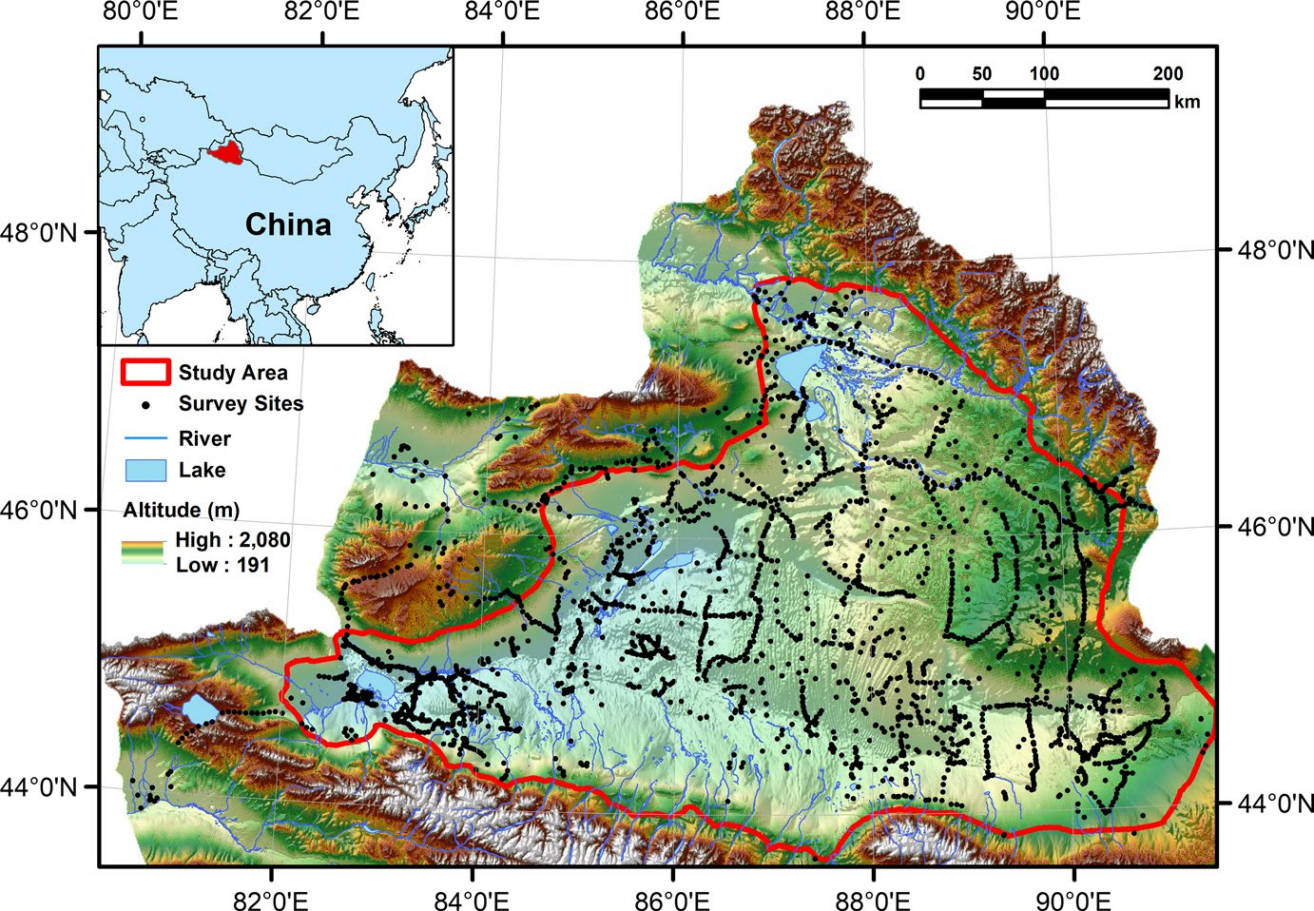
Location of the study area and the 2,516 survey sites. The color map shows the elevation, rivers and lakes of the Junggar Basin

The Junggar Basin (Figure 2) is located in the northern part of Xinjiang province in northwestern China and covers an area of ca. 187,800 km^2^ (Tang et al., 2008). In the center of the basin is the Gurbantunggut Desert, 97% of which is comprised of fixed and semi‐fixed sand ridges. The basin is surrounded by the Altai Mountains in the north and the Tianshan Mountains in the south, and the elevation increases from southwest to northeast. The whole area is characterized by a temperate continental arid or semi‐arid climate with an annual mean temperature (MAT) of 1.3–9.8°C and mean annual precipitation (MAP) of 100–230 mm. The dominant species in this region include *H. ammodendron*, *H. persicum*, *A. aphylla*, *C. mongolicum*, various annual herbs, and several ephemeral plant species.

### 2.2 Species distribution data

We conducted extensive field investigations in northern Xinjiang during the summer (May to September) of 2015 and 2016. We investigated the dominant species of the herbaceous and woody layer in each sampling point, which is distributed every 1–2 km along the major roads in this region. The distance from the point to the road is far enough to avoid possible human disturbance. Elevation and geographical coordinates of each sampling point were also recorded. At the same time, we also took 2–4 photographs in each sampling point with a Canon digital camera. Plant species that were difficult to recognize in the field were collected and sent back to the laboratory for further recognition. To ensure the accuracy of the information of plants species, one of the authors who has expertise on plants in this region cross‐referenced all the records and samples of plant species to Flora of China (http://frps.iplant.cn) and Keys to the Higher Plants of Xinjiang (Hudabaierdi & Xu, 2000). Overall, we investigated 2,516 sites, collected 273 plant samples and took 8,356 photographs both inside and out of the Junggar Basin. These points were distributed across the entire region and represented all of the major types of ecological communities (Figure 2). The presence (i.e., 1) and absence (i.e., 0) of each species in each point were identified to construct a database containing 279,276 records for 111 species. For this study, we used the presence and absence data recorded for the four studied species (*H. ammodendron*, *A. aphylla*, *C. mongolicum*, and *P. euphratica*). The number and distribution of these records were presented in Table 1 and Figure S1. For each species, all the 2,516 presence and absence records both inside and out of the Junggar Basin were used to calibrate the SDM, but the model projections were made only within the basin.

**Table 1:** Number of presence records and model performance for each species

Species	Number of presence records^a^	AUC of all models^b^	TSS of all models^c^	% of selected models^d^	Best performing algorithms^e^
*Haloxylon ammodendron*	1,338	0.770 ± 0.057	0.434 ± 0.072	29	RF, GBM, GAM
*Anabasis aphylla*	385	0.835 ± 0.047	0.592 ± 0.066	84	RF, GBM, MaxEnt
*Calligonum mongolicum*	157	0.782 ± 0.050	0.497 ± 0.078	43	GBM, MaxEnt, RF
*Populus euphratica*	118	0.943 ± 0.039	0.842 ± 0.064	99	RF, GBM, MARS

a Number of species presence records from the 2,516 sampling points.

b The standard deviation from the mean AUC of all models (10 modeling algorithms × 10 replicates).

c The standard deviation from the mean TSS of all models (10 modeling algorithms × 10 replicates).

d The percentage of models selected based on the criterion of AUC > 0.8 and TSS > 0.45.

e The top three algorithms with the highest mean AUC scores of 10 replicates.

### 2.3 Environmental variables

We used 19 bioclimatic variables derived from the monthly temperature and precipitation values, which represent annual trends, seasonality, and extreme or limiting environmental factors (Fick & Hijmans, 2017). Current climate values are the average for the years 1970–2000. To predict future distributions of the four species in Junggar Basin, we also used projected future climate in the medium (2050s, mean of 2030–2060) and long term (2070s, mean of 2060–2080). Here, we considered three Representative Concentration Pathways (RCPs), including an optimistic (RCP2.6), a moderate (RCP4.5), and a pessimistic scenario (RCP8.5) proposed by IPCC Fifth Assessment Report (Cubasch et al., 2013). As large variability exists in the projected future climate by different general circulation models (GCMs; Goberville, Beaugrand, Hautekeete, Piquot, & Luczak, 2015), we selected the projections from seven GCMs from Coupled Model Intercomparison Project Phase 5 (CMIP5), including BCC‐CSM1‐1 (Beijing Climate Center, China), CCSM4 (National Center for Atmospheric Research, USA), GISS‐E2‐R (NASA Goddard Institute for Space Studies, USA), HadGEM2‐ES (Met Office Hadley Centre, UK), IPSL‐CM5A‐LR (Institut Pierre‐Simon Laplace, France), MRI‐CGCM3 (Meteorological Research Institute, Japan), and NorESM1‐M (Norwegian Climate Centre, Norway). All the raster maps of current and future bioclimatic variables at 30‐s resolution were downloaded from the WorldClim database (http://worldclim.org/version2; Fick & Hijmans, 2017).

Besides climatic variables, topographical variables were also included in our models. First, slope and altitude at a spatial resolution of 30 m were calculated based on the digital elevation model (DEM) generated by the Shuttle Radar Topography Mission (SRTM), which were obtained from the United States Geological Survey (USGS) Earth Resources Observation and Science (EROS) Center (USGS, 2015). Second, the distances to fresh and salty waterbodies were also used as predictors in the models due to the following reason. Groundwater level is one of the decisive factors for plant growth in this region but the data are difficult to obtain. The conditions of groundwater are closely related to the distance to a waterbody (Aishan, Betz, Halik, Cyffka, & Rouzi, 2018); hence, distance to waterbody has been regarded as a important alternative for groundwater level in vegetation mapping. For instance, Levick and Rogers (2011) have used distance from river as an environmental predictor when analyzing vegetation dynamics in African savannah, and Kong, Sun, Chen, Yu, and Tian (2010) have accurately mapped vegetation patch distribution change in Tarim river basin using distance to waterbody as a predictor. Considering the factors above, we included the distance to waterbody in our models. We computed the distance to fresh and salty waterbodies through the following eikonal equation (Equation ((1))) solved by the fast marching method (FMM; Sethian, 1996).

(1) $$\left|\nabla d\left(x\right)\right|={\left.1,\;\text{subjected}\;\text{to}\;d\left(x\right)\right|}_{\bar{\Omega }}=0$$

where *d* (*x*) is the distance of any points in the study area outside of the waterbodies to the waterbodies; Ω represents the waterbodies, and $\bar{\Omega }$ is the boundary of the waterbodies (Zhang et al., 2019). High‐resolution water masks were collected from JRC Global Surface Water Mapping Layers, v1.0 on Google Earth Engine (Pekel, Cottam, Gorelick, & Belward, 2016).

To make future predictions, we assumed the five topographical variables to be stationary. All the environmental variables mentioned above were resampled to 1 km spatial resolution using the nearest neighbor method in ArcGIS 10.3.

Several environmental variables were strongly correlated with each other. To mitigate the impacts of collinearity of predictors on model performance, we applied a Pearson correlation threshold of |*r*| < .70 (Dormann et al., 2013) to select the variables used to calibrate the models (see Table S1). Specifically, only one variable from any pair of strongly correlated variables (i.e., |*r*| ≥ .70) was retained. The decision on which variable to retain was based on literature (Zhang & Chen, 2002; Zhang, Liu, Zhang, & Sun, 2016) and our empirical knowledge. Finally, eight environmental variables (Table 2) were used for the SDM calibrations.

**Table 2:** List of eight environmental predictors for SDM development

Abbreviation	Variable	Unit
MAT	Mean annual temperature	°C
MDR	Mean diurnal range	°C
ISO	Isothermality	%
MTCM	Min temperature of coldest month	°C
PWM	Precipitation of wettest month	mm
PCQ	Precipitation of coldest quarter	mm
DIST_F	Distance to fresh waterbody	km
DIST_S	Distance to salty waterbody	km

### 2.4 Calibration and evaluation of species distribution models

We conducted the modeling process using the “biomod2” package (ver. 3.3‐18; Thuiller, Georges, & Engler, 2014) in R (ver. 3.5.0; R Core Team, 2018). For each species, we used ten modeling algorithms embedded in the biomod2 package: generalized linear models (GLM), boosted regression trees (GBM), generalized additive model (GAM), classification tree analysis (CTA), artificial neural network (ANN), surface range envelop or BIOCLIM (SRE), flexible discriminant analysis (FDA), multiple adaptive regression splines (MARS), random forests (RF), and maximum entropy (MaxEnt). These algorithms were chosen because they have shown good ability to predict current species distribution and have been widely used in ecological modeling (Hamid et al., 2019; Lin & Chiu, 2019; Thuiller, 2003). Combining the predictions of individual algorithms to make ensemble prediction provides results that are more robust and also enables the assessment of the uncertainty generated from the modeling procedure.

Each modeling algorithm was calibrated using data from a random selection of 75% of the input dataset and tested using the remaining 25% data. To avoid the possible bias from the data split, this process was repeated 10 times for each model algorithm. The relative performance of the 100 models (10 modeling algorithm × 10 replicates) for each species was evaluated using the area under the receiver operating characteristic curve (AUC; Hanley & McNeil, 1982) and true skill statistic (TSS; Allouche, Tsoar, & Kadmon, 2006). As TSS is a threshold‐dependent evaluation metric, the threshold that maximized the sum of sensitivity (true positive rate) and specificity (true negative rate) was used (Liu, White, & Newell, 2013). Higher AUC or TSS scores represent better model performance. Following previous studies (Lasram et al., 2010; Wang, Liu, et al., 2018), AUC scores between 0.7–0.8 were classified as “fair” and 0.8–1 as “good,” TSS scores between 0.4–0.6 were classified as “fair” and 0.6–1 as “good.” Only models with AUC scores higher than 0.8 and TSS scores higher than 0.45 were selected and used to predict potential distribution for given species.

The outputs of models were converted into binary maps of presences and absences using a threshold that maximized the sum of sensitivity and specificity (Fielding & Bell, 1997). For the current projection, all the binary maps predicted by the selected models were integrated into a “consensus map” by calculating the percentage of presence of a given species in a cell (see Figure S2). The consensus map was then converted to a binary distribution map following the approach proposed by IPCC‐AR5 (Mastrandrea et al., 2011) to treat the uncertainty and “likelihood” of predictions. Specifically, the grid cells where more than 66% of the models predicted presence of a given species were regarded as the potential distribution range or suitable habitat of this species. Similarly, the consensus map of future projections for a given species under each RCP scenario in each period (Figures S3–S6) was generated from the projections of all selected models based on all GCMs and was converted to binary distribution maps using the same threshold of 66%. In this way, we produced the potential distribution maps for each species under the current and future climates; hence, the changes in potential distribution range could be analyzed. Specifically, the area that was not suitable for a species currently but predicted to be suitable in the future climate scenarios was identified as “habitat gain” and vice versa “habitat loss.”

We further evaluated the relative importance of different climatic variables in shaping the distribution of each species using the biomod2 package (Gama, Crespo, Dolbeth, & Anastacio, 2017). The importance of a variable was calculated as 1 minus the correlation coefficient between the outputs of two models in which the original and shuffled values of the focal variable were used, respectively. The calculation of the importance of a given variable was repeated three times, and the final importance value of this variable was calculated as the mean of the three outputs. High importance values suggest that the shuffle of the focal variable could significantly change model outputs; hence, the focal variable has strong influence on the model.

## 3 RESULTS

### 3.1 Model performance and climatic variable importance

All calibrated models for the four species performed well (Table 1). The mean AUC scores of all models ranged from 0.770 (*H. ammodendron*) to 0.943 (*P. euphratica*) and the mean TSS scores of all models ranged from 0.434 (*H. ammodendron*) to 0.842 (*P. euphratica*), respectively. These results suggest that these modes had strong predictability on the potential distribution of the studied species. Across different modeling algorithms, RF and GBM displayed better performance than did the others.

**Figure 3:**
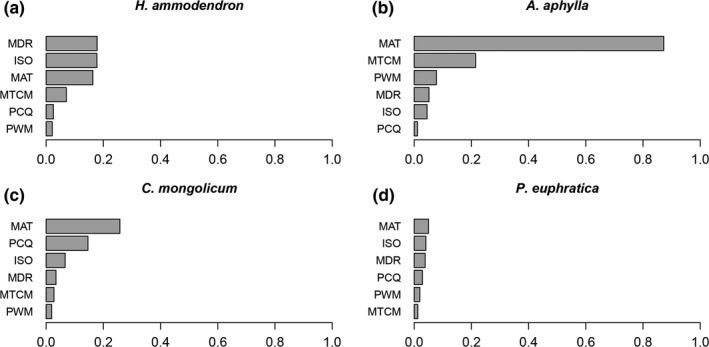
Mean variable importance for *Haloxylon ammodendron* (a), *Anabasis aphylla* (b), *Calligonum mongolicum* (c), and *Populus euphratica* (d). ISO, Isothermality; MAT, Mean Annual Temperature; MDR, Mean Diurnal Range; MTCM, Min Temperature of Coldest Month; PCQ, Precipitation of Coldest Quarter; PWM, Precipitation of Wettest Month

The most important climate variables influencing species distributions differed significantly across the four species (Figure 3). For *H. ammodendron*, the most important variables were temperature‐related variables (i.e., MDR, ISO, MAT, MTCM), while precipitation‐related variables had only limited influences. MAT displayed an exceedingly strong influence in the prediction of *A. aphylla* distribution with an importance score of 0.872, while other variables showed only minor influence. MAT and PCQ were key variables for the prediction of *C. mongolicum*. For *P. euphratica*, it seems that climatic variables are of little importance to its potential distribution.

### 3.2 Potential distribution change

**Figure 4:**
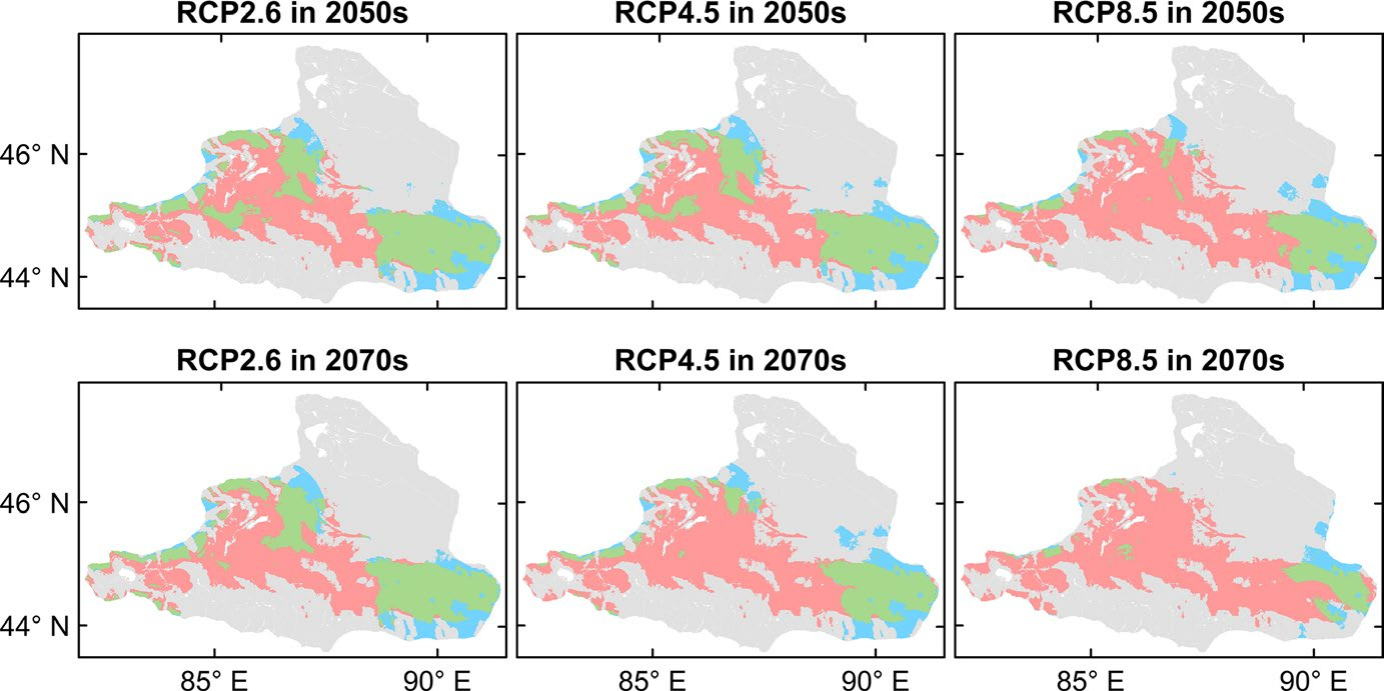
Predicted range change of *Haloxylon ammodendron* in the Junggar Basin under three climate change scenarios (i.e., optimistic—RCP2.6, moderate—RCP4.5, and pessimistic—RCP8.5) and two periods (i.e., 2050s and 2070s). Green, blue, and red colors represent overlap of current and future predicted ranges, potential range expansion, and potential range contraction, respectively

**Figure 5:**
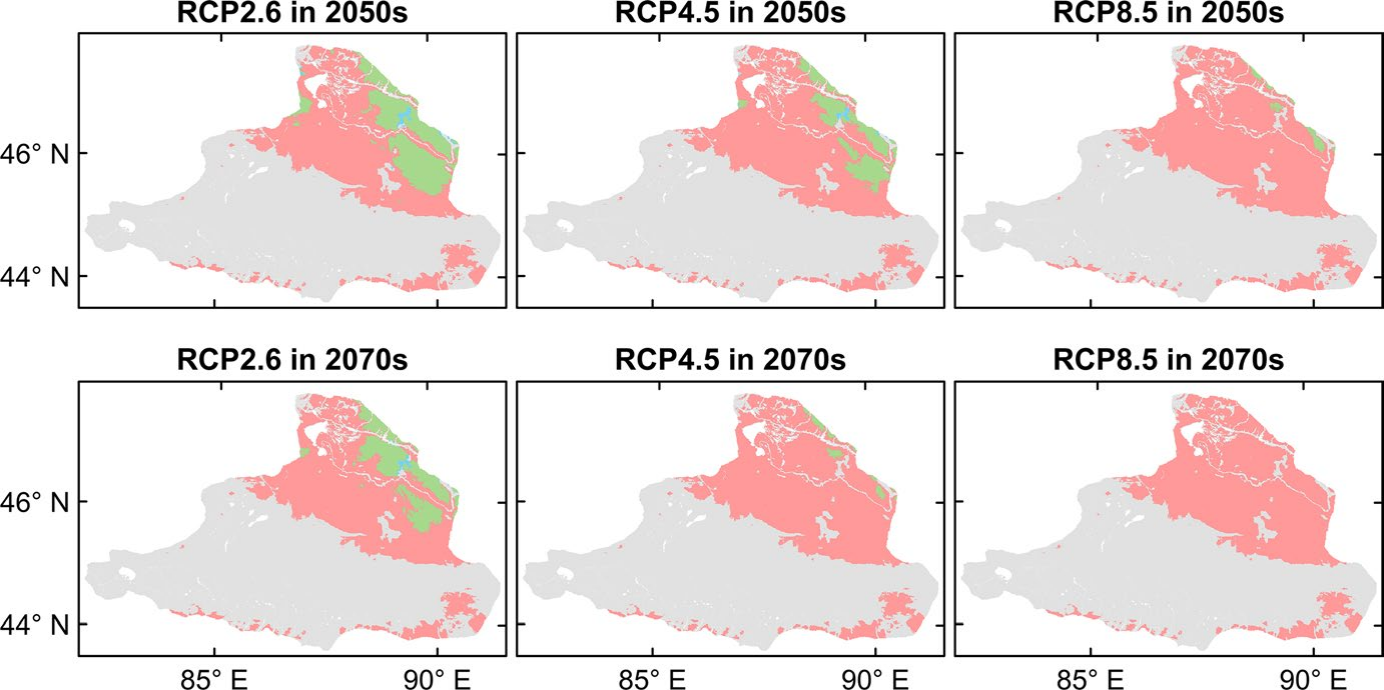
Predicted range change of *Anabasis aphylla* in the Junggar Basin under three climate change scenarios (i.e., optimistic—RCP2.6, moderate—RCP4.5, and pessimistic—RCP8.5) and two periods (i.e., 2050s and 2070s). Green, blue, and red colors represent overlap of current and future predicted ranges, potential range expansion, and potential range contraction, respectively

**Figure 6:**
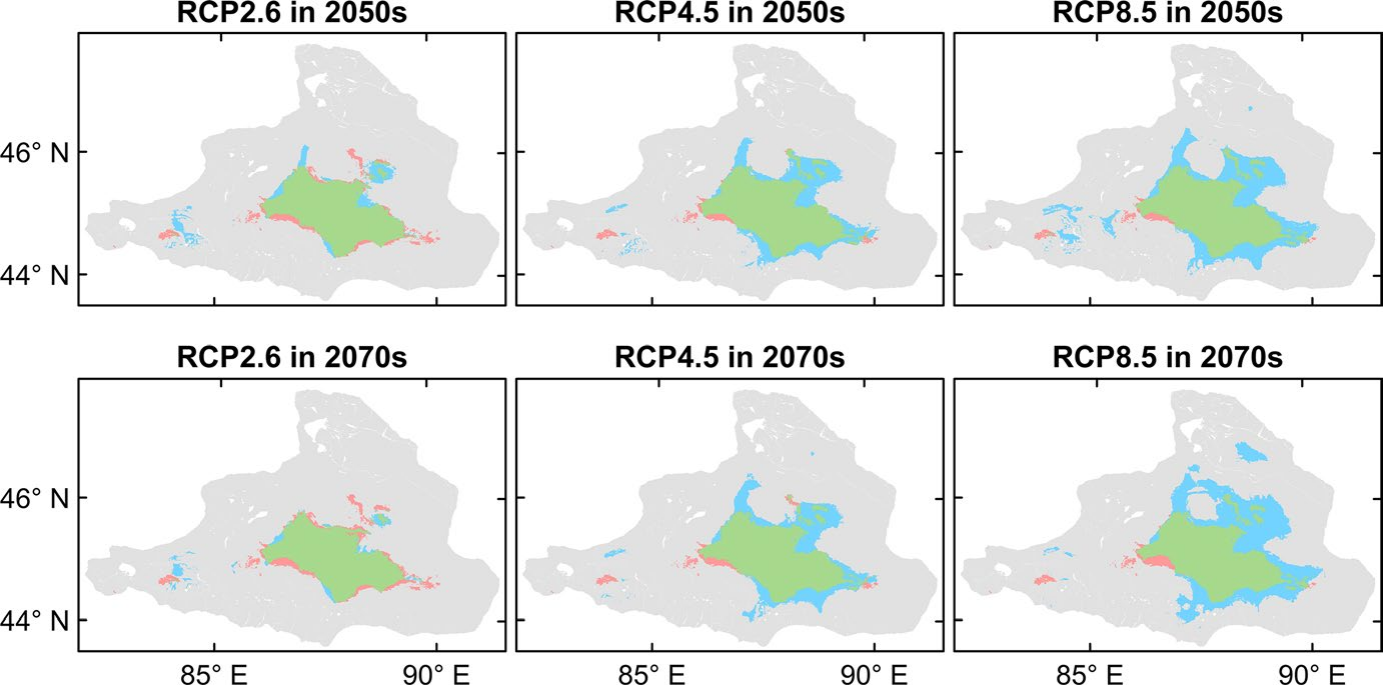
Predicted range change of *Calligonum mongolicum* in the Junggar Basin under three climate change scenarios (i.e., optimistic—RCP2.6, moderate—RCP4.5, and pessimistic—RCP8.5) and two periods (i.e., 2050s and 2070s). Green, blue, and red colors represent overlap of current and future predicted ranges, potential range expansion, and potential range contraction, respectively

**Figure 7:**
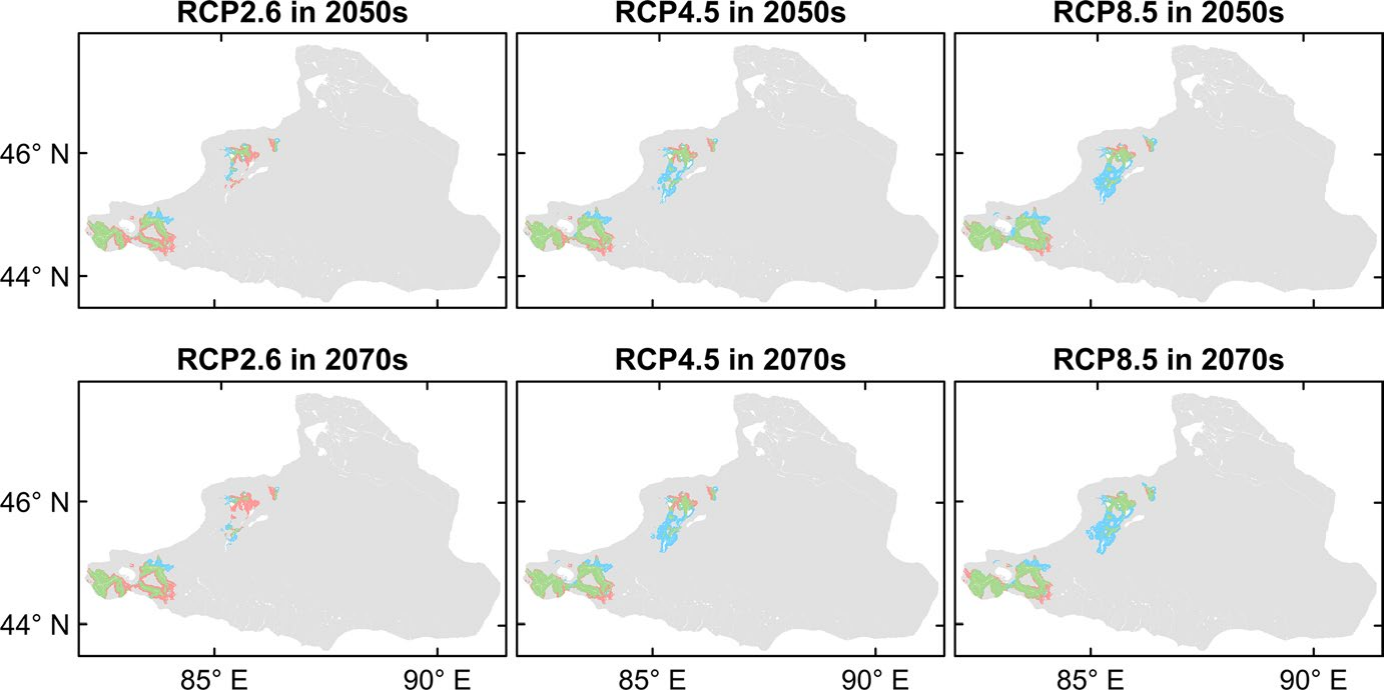
Predicted range change of *Populus euphratica* in the Junggar Basin under three climate change scenarios (i.e., optimistic—RCP2.6, moderate—RCP4.5, and pessimistic—RCP8.5) and two periods (i.e., 2050s and 2070s). Green, blue, and red colors represent overlap of current and future predicted ranges, potential range expansion, and potential range contraction, respectively

**Figure 8:**
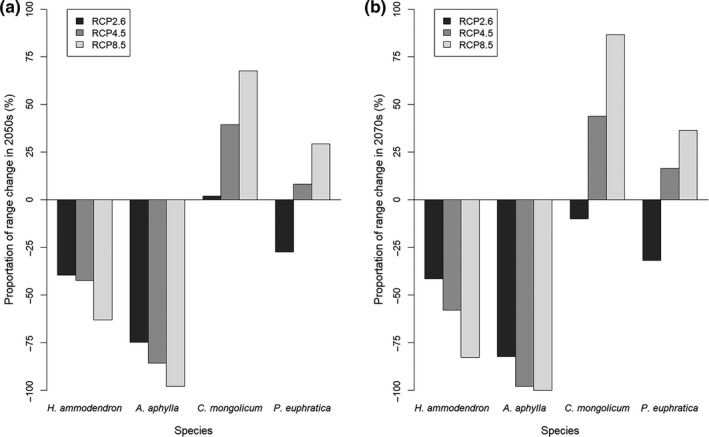
Proportion of species range change in the Junggar Basin in 2050s (a) and 2070s (b) under three climate change scenarios (i.e., optimistic—RCP2.6, moderate—RCP4.5, and pessimistic—RCP8.5), calculated by proportion of habitat gain minus proportion of habitat loss

The four species differed in the extent or direction of predicted range change among the future climate scenarios and periods (Figures 4, 5, 6, 7, 8). *Haloxylon ammodendron* and *A. aphylla* were predicted to suffer from potential range contraction in all future climate scenarios and periods, and the contraction increased with the climate scenario severity. Specifically, *H. ammodendron* was mainly threatened in the western part of its current distribution range, while the eastern part was likely to be relatively stationary in the future (Figure 4). The potential distribution of *A. aphylla* exhibited an obvious northward retreat. The southern part of its current distribution range was expected to contract extensively, and suitable habitats were likely to remain only in the mountainous regions of northeastern Junggar Basin under climate scenarios of RCP4.5 and RCP8.5 (Figure 5). In contrast, suitable habitats of *C. mongolicum* and *P. euphratica* were expected to expand in moderate and pessimistic climate scenarios. In RCP2.6, both species exhibited minor contraction except for *C. mongolicum* in the 2050s, where slight expansion was predicted. The range change of *C. mongolicum* mainly occurred in the margin of the current distribution range with slight contraction in the RCP2.6 scenario and considerable expansion in RCP4.5 and RCP8.5 (Figure 6). The range change of *P. euphratica* was less significant compared to the other species and mainly occurred in the northern part of its current distribution (Figure 7). Generally, the dynamics of range change identified in the medium term (2050s) were reinforced in the long term (2070s).

## 4 DISCUSSION

### 4.1 Different responses of species to climate change

Over the past few decades, the increase of temperature and precipitation has been recorded in the northwestern China (Li, Chen, Shi, Chen, & Li, 2013). We calculated the mean changes in the six climate variables between current and future climates across the seven GCMs and found that both temperature and precipitation would continue to increase in the future in most parts of the Junggar Basin (see Figures S7–S12). However, our results showed that the four studied species tended to respond to climate change differently.

*H. ammodendron*, the dominant plant species of the Junggar Basin's desert ecosystem, was predicted to lose 39.6%–63.0% of its current suitable habitat in the 2050s and 41.5%–82.8% in the 2070s. Our prediction is consistent with that of Wu, Lu, and Zhou (2010) but contrary to Ma, Wei, Li, Luo, and Sun (2017). Ma et al. used a limited amount of species occurrence data obtained from literature and only one algorithm, which may explain their different results from ours (Buisson, Thuiller, Casajus, Lek, & Grenouillet, 2010). The range contraction of *H. ammodendron* might be closely related to the lack of regeneration under a future warmer climate. Although *H. ammodendron* is drought‐tolerant, studies have found that its seedlings' survival and natural regeneration depends heavily on the soil moisture replenished by the melted snow layer in early spring of the region (Tian, Tashpolat, & Li, 2014). The rapid warming trend in this region, especially the rapid temperature rise in spring, may cause the soil to dry out fast, so that seedlings may fail to survive (Huang, Xiang, Li, & Xu, 2009). These findings have been supported by empirical evidence. Huang, Li, and Yuan (2008) had observed massive mortality of seedlings and degradation of communities of *H. ammodendron* in the southern edge of the Junggar Basin, and the deficiency of soil water caused by high temperatures in spring had been considered to be one of the major causes.

Similar to *H. ammodendron*, the potential habitat loss of *A. aphylla* also increased with an increase in severity of climate change scenario. In the extreme case of RCP8.5 scenario in the 2070s, 100% of habitat loss was predicted, implying that the Junggar Basin would not be suitable for the distribution of *A. aphylla* anymore. We can also infer from the range change maps that the potential distribution of *A. aphylla* will be limited to the mountainous area in the northeastern Junggar Basin where the climate is colder and wetter. The germination process of the seedlings of *A. aphylla* is very similar to that of *H. ammodendron*, depending heavily on the spring temperature and the water supply of melting snow (Chu, 2015). Previous studies on the seed germination of *A. aphylla* have found that seeds of this species germinated better in a cool and wet early spring, while high temperatures restricted the germination (Chu, 2015). Thus, it is reasonable to assume that it may experience the same decline in regeneration as *H. ammodendron* in the warmer future. In addition, among the results of variable importance evaluation, MAT was the single most influencing variable on the distribution of *A. aphylla* with an importance score of 0.872. Wang, Wen, Zhang, and Zhang (2018) also reported similar results in the predictions of the potential distribution of *Anabasis* species. These findings together indicated that *A. aphylla* may be very sensitive to the change of temperature.

Contrary to *H. ammodendron* and *A. aphylla*, *C. mongolicum* seems to be a “winner” under future climate scenarios, with its suitable habitat expanding by 1.87%–67.6% in the 2050s and −9.94% to 86.54% in the 2070s. The expansion may be explained by the promotion of a higher temperature and precipitation amount to the germination and growth of *C. mongolicum*. Previous studies have found that low temperatures can significantly delay the onset of germination of *Calligonum* species (Ren, Jin, & Ling, 2005). The low capacity of *Calligonum* species to germinate at a lower temperature is consistent with their more frequent occurrences in sandy regions with high soil temperatures in summer (Eziz, 2018). Moreover, *C. mongolicum* has a quite different root morphology and water use strategy from *H. ammodendron*. The root system of *H. ammodendron* could penetrate up to 10 m below the soil surface and reach the groundwater, which ensures its survival during severe drought stress and makes it less sensitive to the change of precipitation (Xu & Li, 2008). In contrast, *C. mongolicum* has less developed taproots (about 3 m deep) but more advanced lateral roots (about 10–20 m in a horizontal direction; Li, Lei, Zhao, Xu, & Li, 2015), making it depend more on surface water and more susceptible to precipitation change than *H. ammodendron* (Li et al., 2017). Simulated precipitation change experiments also found that the seedlings of *C. mongolicum* need more water to survive than other desert species and prefer a water supply condition of more than 88 mm of precipitation (Li & Zhao, 2006). Considering the two factors mentioned above, we could infer that the current distribution of *C. mongolicum* may be mainly limited by low temperature and precipitation, which is also consistent with our results of the variable importance evaluation. The future increase of temperature and precipitation in this region is, hence, likely to relieve this limitation and trigger the expansion of its potential distribution. Our prediction is consistent with that of Hamit, Abdushalih, Xu, and Jiesisi (2018) and Liu, Feng, and Guan (2016) in suggesting the potential expansion of *C. mongolicum* in other regions. Nevertheless, in the RCP2.6 scenario of the 2070s, a range contraction of 9.94% was predicted for *C. mongolicum*. This result is in line with the findings of Zhang (2018), who predicted the suitable areas of 10 plant species in the arid region of northwestern China are expected to shrink under a low‐emission scenario but expand under a higher‐emission scenario. The possible explanation is that the higher evaporation rate resulting from increased temperature may neutralize the effects of a precipitation increase under a low‐emission scenario, causing the loss of suitable habitat.

The variable importance evaluation showed that climatic variables have only marginal influence on the distribution of *P. euphratica*. This result corresponds to our field observation and other research, for *P. euphratica* forest is intrazonal vegetation in the Junggar Basin and, basically, only distributed along the desert river bank (Huang, 1990). The water source of the *P. euphratica* forest originates mainly from deep subsoil water and groundwater, which is commonly recharged by a river streamflow and flood (Si, Feng, Cao, Yu, & Zhao, 2014). Therefore, *P. euphratica* is less susceptible to climatic variation but more susceptible to distance from a waterbody (Aishan et al., 2018). In this context, as we assumed the distance to waterbody remains stationary in the future, the potential distribution of *P. euphratica* is unlikely to experience a massive transformation, which was also implied in our prediction result.

### 4.2 The consequences of species range change

To fix sand dune and combat desertification, *H. ammodendron*, *C. mongolicum*, and other shrub species have been widely used in afforestation in the drylands of northwestern China (Yu & Wang, 2018). Though some considered *H. ammodendron* a better afforestation species than *C. mongolicum* due to its stronger drought tolerance (Xu, Ji, Jin, & Zhang, 2017; Yao, Yan, & Yang, 2016), our prediction result showed that the suitable habitat of *H. ammodendron* may contract substantially in the Junggar Basin, while the suitable habitat of *C. mongolicum* is likely to expand. The different changes in climate suitability of these two species implies that policymakers may need to reconsider the selection and combination of the plant species to achieve better afforestation results in this region under future climate changes.

In arid regions, shrubs usually play an important role in modifying microclimate, supporting local plant communities, and determining landscape diversity (Segoli, Ungar, Giladi, Arnon, & Shachak, 2012). For example, they can form the so‐called “fertile islands” under their canopies, increasing water and nutrient content in the soil in these fertile islands, which are primary limiting factors for the structure, production, and dynamics of vegetation in arid ecosystems (Schlesinger, Raikes, Hartley, & Cross, 1996). However, the development of fertile islands and their effects on soil and other plants may be greatly species‐dependent due to different morphological characteristics and physiological processes among shrub species (Li, Zhao, Zhu, Li, & Wang, 2007). Zhang, Zhao, Yang, Zhao, and Wang (2016) even found contrasting effects of *H. ammodendron* and *C. mongolicum* on understory species. Therefore, different distribution changes of dominant shrubs of the Junggar Basin may also influence the dependent organisms and ecosystems that are connected to these shrub species, causing them to relocate, expand, or deteriorate.

*P. euphratica* is the dominant species of the riparian forests in the Junggar Basin. As a distinct ecotone between rivers and the surrounding dryland, the riparian zone is of tremendous importance in the ecological and hydrological processes of arid regions (Newman et al., 2006 ). Although our prediction implied that the potential distribution of *P. euphratica* may not change as significantly as other species do in the future, climate change could still have an apparent impact on the hydrology condition of the riparian zone, which is a main driving factor of *P. euphratica* riparian forests (Keram et al., 2019). In addition, the degradation of the *P. euphratica* forest has been reported to cause oasis desertification and biodiversity loss in the Tarim Basin, a desert zone located in the south of the Junggar Basin (Liu, Chen, Chen, Zhang, & Li, 2005). Therefore, more studies are needed to evaluate the effects of climate change on *P. euphratica* distribution through changes in hydrology conditions.

### 4.3 Limitation and prospect

Based on comprehensive field investigation data and the ensemble SDM method, we revealed the possible change in potential distribution of the dominant woody plant species in Junggar Basin. However, we did not take into account some other important factors, besides climatic and topographical ones, in the model calibration. For example, human activities like water overexploitation can significantly affect the streamflow of a waterbody and, consequently, the living conditions of the riparian *P. euphratica* forest (Hao, Chen, Xu, & Li, 2008; Xu, Ye, Song, & Chen, 2007). Therefore, assuming that the waterbody would remain the same in the future may lead to an overly optimistic estimation of the habitat change of *P. euphratica*.

Our investigation range is limited to northern Xinjiang, which is not large enough to cover the present full habitat ranges of the studied species. Therefore, the prediction of our model could potentially be biased by the incomplete bioclimatic niche space sampled (Wolmarans, Robertson, & van Rensburg, 2010). However, as the Junggar Basin (or northern Xinjiang) is a region with a relatively clear biogeographic boundary and unique bioclimatic conditions and our sampling points cover the full range of this area, we believe that our prediction provides useful information on the potential distribution of the species within this region (Barve et al., 2011).

Despite the limitations mentioned above, considering the lack of studies investigating the effects of climate change on plant distribution in the Junggar Basin and other drylands in the world, this study is valuable at the current stage by providing a better understanding of dryland plant species' responses to climate change, as well as a useful reference to dryland conservation and restoration. Further studies are still needed to investigate a wider habitat range and incorporate other important factors like human activities into the estimation of possible changes of dryland plant species distribution and to facilitate the conservation planning and policymaking in drylands under future climate change scenarios.

## CONFLICT OF INTEREST

None declared.

## AUTHOR CONTRIBUTIONS

The authors have an interest in the ecology and conservation of the dryland ecosystems and plant species in northwestern China. A.E. designed and conducted the field investigation; J.X. and A.E. led the analyses and writing; H.Z. contributed to concept development and data analyses; Z.W., Z.T., and J.F. provided technical guidance to the study. All authors contributed to and approved the final manuscript.

## Data Availability

Environmental variables used as predictor in this study are publicly available and sourced in the main text of this paper. The distribution data of the four studied species were archived in Dryad (https://doi.org/10.5061/dryad.qnk98sfbg).
