# Supplementary material for: Responses of four dominant dryland plant species to climate change in the Junggar Basin, northwest China
Source: Ecol Evol. 2019 Nov 11;9(23):13596–607. doi: 10.1002/ece3.5817 (PMC6912881; doi:10.1002/ece3.5817)

**
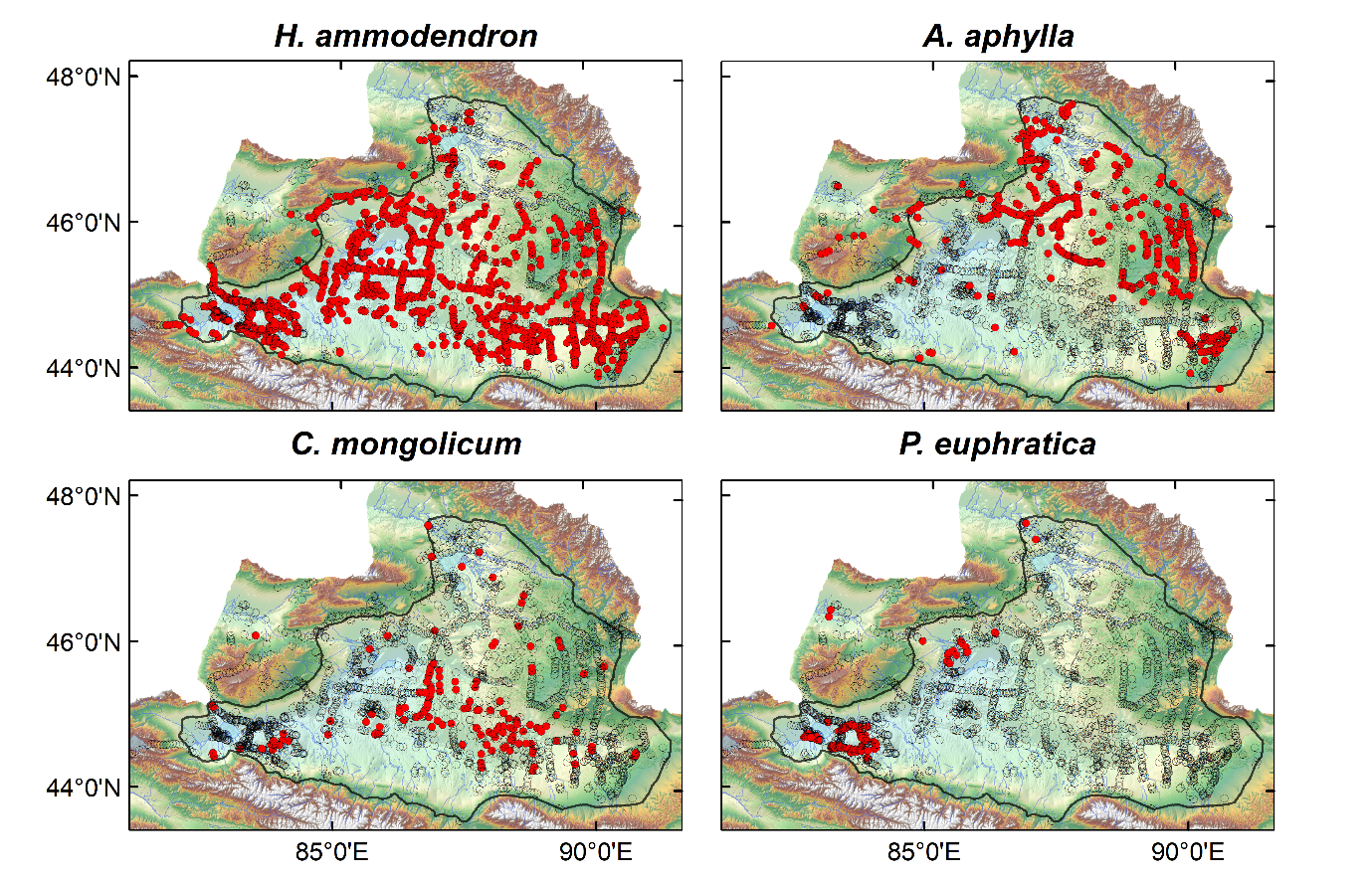
Figure S1** Distribution of the present and absent points for *Haloxylon ammodendron*, *Anabasis aphylla*, *Calligonum mongolicum*, and *Populus euphratica*. Red circles represent present points while blank circles represent absent points.

**Table S1** Pearson correlation among 23 environmental variables.

|  | BIO1 | BIO2 | BIO3 | BIO4 | BIO5 | BIO6 | BIO7 | BIO8 | BIO9 | BIO10 | BIO11 | BIO12 | BIO13 | BIO14 | BIO15 | BIO16 | BIO17 | BIO18 | BIO19 | ALT | SLP | DIST_  F |
| --- | --- | --- | --- | --- | --- | --- | --- | --- | --- | --- | --- | --- | --- | --- | --- | --- | --- | --- | --- | --- | --- | --- |
| BIO2 | 0.088 |  |  |  |  |  |  |  |  |  |  |  |  |  |  |  |  |  |  |  |  |  |
| BIO3 | -0.507 | 0.439 |  |  |  |  |  |  |  |  |  |  |  |  |  |  |  |  |  |  |  |  |
| BIO4 | 0.561 | 0.234 | -0.760 |  |  |  |  |  |  |  |  |  |  |  |  |  |  |  |  |  |  |  |
| BIO5 | 0.877 | 0.347 | -0.583 | 0.852 |  |  |  |  |  |  |  |  |  |  |  |  |  |  |  |  |  |  |
| BIO6 | -0.062 | -0.343 | 0.520 | -0.828 | -0.499 |  |  |  |  |  |  |  |  |  |  |  |  |  |  |  |  |  |
| BIO7 | 0.595 | 0.399 | -0.647 | 0.972 | 0.895 | -0.829 |  |  |  |  |  |  |  |  |  |  |  |  |  |  |  |  |
| BIO8 | 0.964 | 0.836 | -0.689 | 0.848 | 0.987 | -0.432 | 0.852 |  |  |  |  |  |  |  |  |  |  |  |  |  |  |  |
| BIO9 | 0.683 | -0.044 | 0.797 | -0.290 | 0.224 | 0.705 | -0.281 | 0.265 |  |  |  |  |  |  |  |  |  |  |  |  |  |  |
| BIO10 | 0.961 | 0.989 | -0.667 | 0.839 | 0.984 | -0.425 | 0.850 | 0.998 | 0.271 |  |  |  |  |  |  |  |  |  |  |  |  |  |
| BIO11 | 0.338 | -0.388 | 0.396 | -0.582 | -0.097 | 0.895 | -0.595 | -0.055 | 0.936 | -0.047 |  |  |  |  |  |  |  |  |  |  |  |  |
| BIO12 | -0.375 | -0.253 | 0.369 | -0.591 | -0.532 | 0.394 | -0.544 | -0.536 | 0.075 | -0.537 | 0.289 |  |  |  |  |  |  |  |  |  |  |  |
| BIO13 | -0.459 | -0.395 | 0.472 | -0.792 | -0.710 | 0.633 | -0.780 | -0.684 | 0.868 | -0.682 | 0.434 | 0.887 |  |  |  |  |  |  |  |  |  |  |
| BIO14 | -0.246 | -0.060 | 0.293 | -0.377 | -0.399 | 0.938 | -0.305 | -0.348 | 0.023 | -0.354 | 0.630 | 0.820 | 0.588 |  |  |  |  |  |  |  |  |  |
| BIO15 | -0.304 | -0.368 | 0.477 | -0.664 | -0.538 | 0.560 | -0.633 | -0.529 | 0.261 | -0.564 | 0.444 | 0.802 | 0.920 | 0.430 |  |  |  |  |  |  |  |  |
| BIO16 | -0.267 | -0.356 | 0.360 | -0.657 | -0.521 | 0.552 | -0.678 | -0.500 | 0.273 | -0.499 | 0.459 | 0.932 | 0.951 | 0.652 | 0.943 |  |  |  |  |  |  |  |
| BIO17 | -0.279 | -0.666 | 0.268 | -0.380 | -0.350 | 0.989 | -0.323 | -0.371 | -0.023 | -0.376 | 0.359 | 0.866 | 0.655 | 0.964 | 0.451 | 0.677 |  |  |  |  |  |  |
| BIO18 | -0.356 | -0.387 | 0.395 | -0.700 | -0.599 | 0.574 | -0.678 | -0.575 | 0.264 | -0.572 | 0.428 | 0.907 | 0.977 | 0.590 | 0.967 | 0.986 | 0.699 |  |  |  |  |  |
| BIO19 | -0.399 | -0.432 | 0.289 | -0.395 | -0.436 | 0.571 | -0.360 | -0.454 | -0.483 | -0.460 | 0.038 | 0.793 | 0.571 | 0.930 | 0.356 | 0.575 | 0.952 | 0.539 |  |  |  |  |
| ALT | -0.865 | 0.075 | 0.774 | -0.733 | -0.869 | 0.366 | -0.709 | -0.877 | -0.201 | -0.863 | 0.055 | 0.449 | 0.581 | 0.262 | 0.588 | 0.420 | 0.292 | 0.504 | 0.255 |  |  |  |
| SLP | 0.881 | 0.039 | -0.589 | 0.628 | 0.823 | -0.859 | 0.624 | 0.873 | 0.444 | 0.865 | 0.409 | -0.570 | -0.622 | -0.366 | -0.537 | -0.488 | -0.396 | -0.559 | -0.450 | -0.860 |  |  |
| DIST_  F | -0.085 | 0.394 | 0.285 | 0.047 | 0.056 | -0.032 | 0.052 | -0.028 | -0.037 | -0.003 | -0.032 | -0.428 | -0.591 | -0.723 | -0.355 | -0.206 | -0.433 | -0.698 | -0.588 | 0.283 | -0.607 |  |
| DIST_  S | -0.495 | 0.490 | 0.558 | -0.207 | -0.308 | -0.034 | -0.830 | -0.410 | -0.295 | -0.401 | -0.207 | -0.284 | -0.985 | -0.886 | -0.236 | -0.344 | -0.252 | -0.286 | -0.094 | 0.580 | -0.367 | 0.367 |

Note: BIO1 = Annual Mean Temperature; BIO2 = Mean Diurnal Range (Mean of monthly (max temp - min temp)); BIO3 = Isothermality (BIO2/BIO7) (* 100); BIO4 = Temperature Seasonality (standard deviation *100); BIO5 = Max Temperature of Warmest Month; BIO6 = Min Temperature of Coldest Month; BIO7 = Temperature Annual Range (BIO5-BIO6); BIO8 = Mean Temperature of Wettest Quarter; BIO9 = Mean Temperature of Driest Quarter; BIO10 = Mean Temperature of Warmest Quarter; BIO11 = Mean Temperature of Coldest Quarter; BIO12 = Annual Precipitation; BIO13 = Precipitation of Wettest Month; BIO14 = Precipitation of Driest Month; BIO15 = Precipitation Seasonality (Coefficient of Variation); BIO16 = Precipitation of Wettest Quarter; BIO17 = Precipitation of Driest Quarter; BIO18 = Precipitation of Warmest Quarter; BIO19 = Precipitation of Coldest Quarter; ALT = Altitude; SLP = Slope; DIST_F = Distance to Fresh Waterbody; DIST_S = Distance to Salty Waterbody;

**
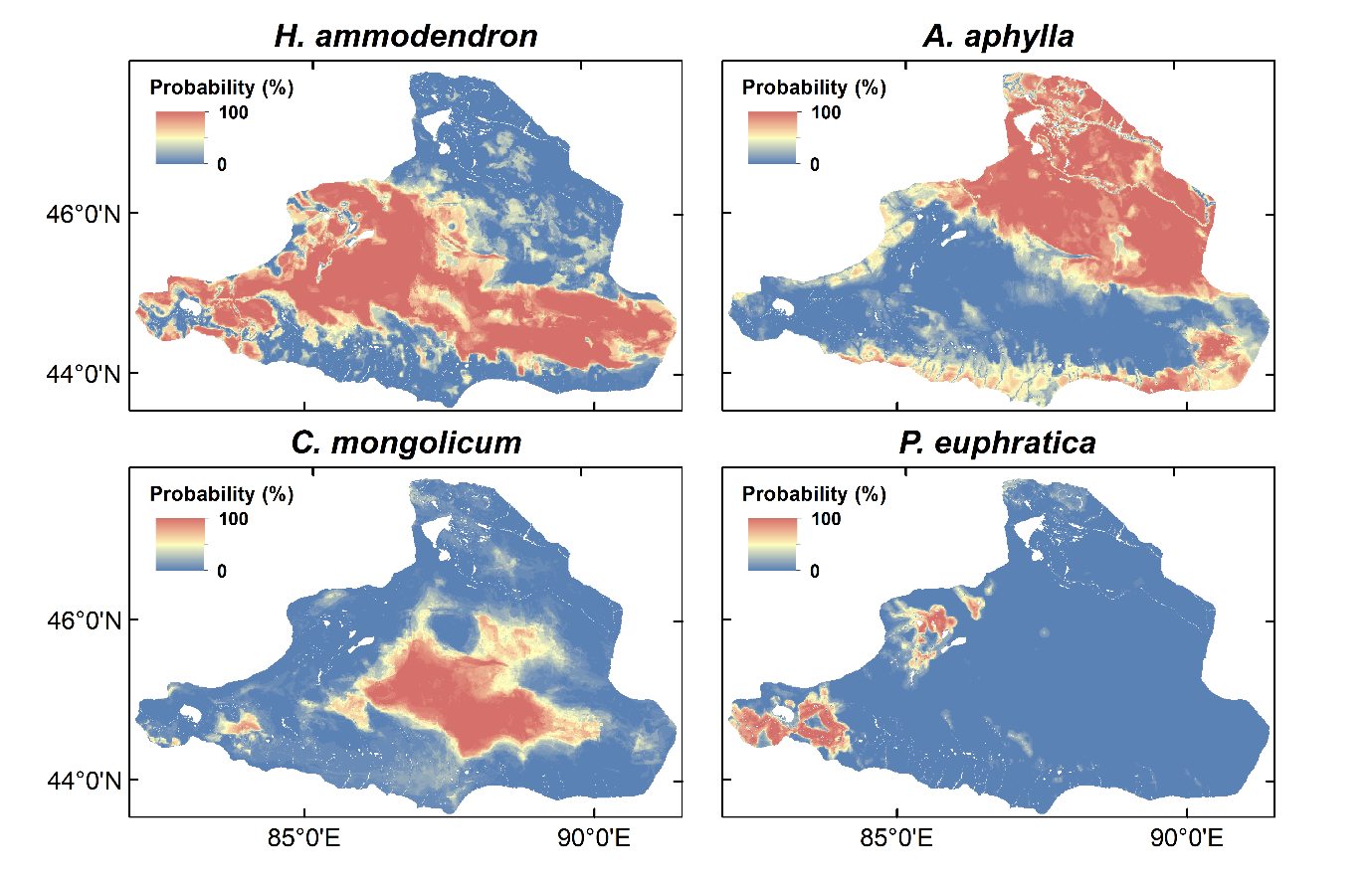
Figure S2** The current consensus maps for *Haloxylon ammodendron*, *Anabasis aphylla*, *Calligonum mongolicum*, and *Populus euphratica*. Raster values are the frequency of predictions of all selected models that indicated a given species was present in each cell. It can be regarded as a measure of habitat suitability.


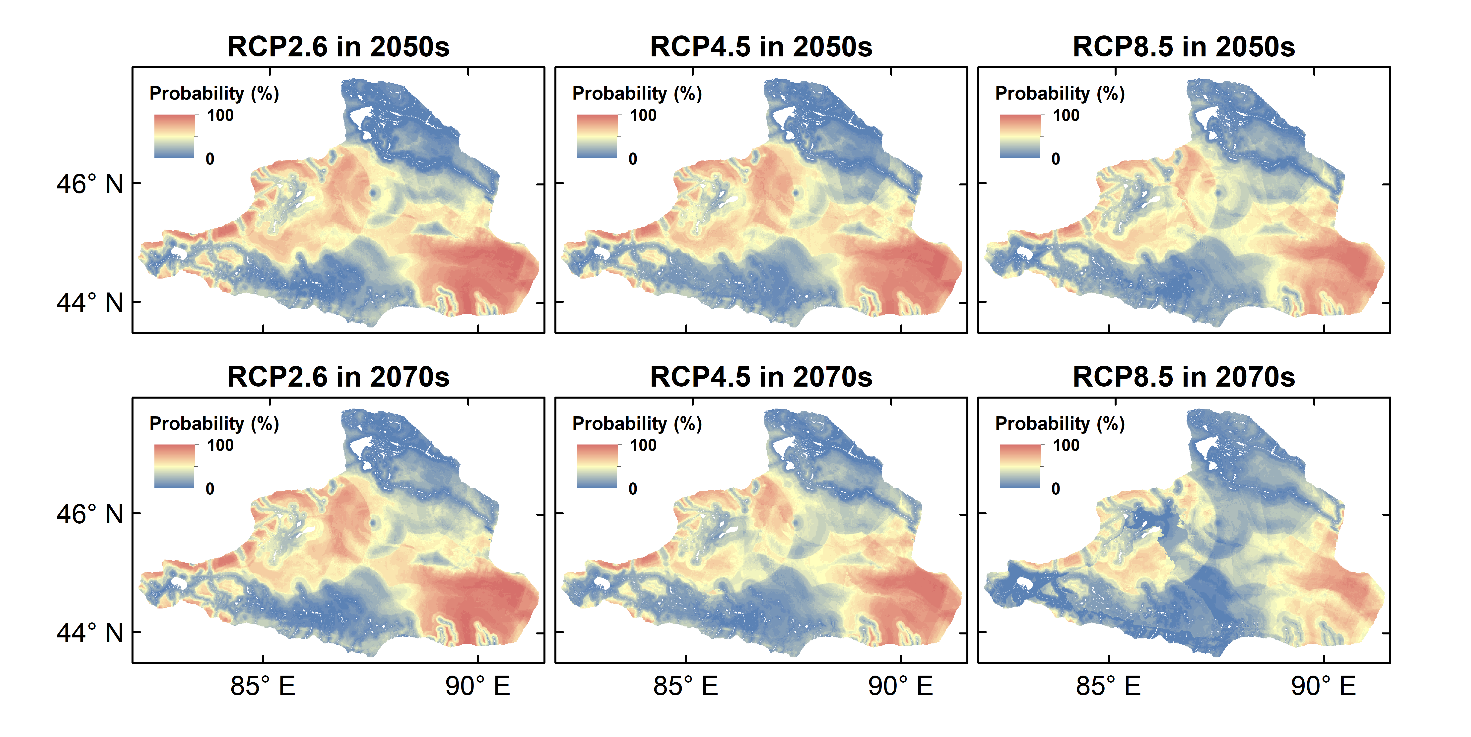
**Figure S3** Predicted future consensus maps of *Haloxylon ammodendron* in the Junggar Basin under three climate change scenarios (i.e. optimistic-RCP2.6; moderate-RCP4.5; pessimistic-RCP8.5) and two periods (i.e. 2050s and 2070s). Each map was generated from the predictions of all selected models based on all GCMs.


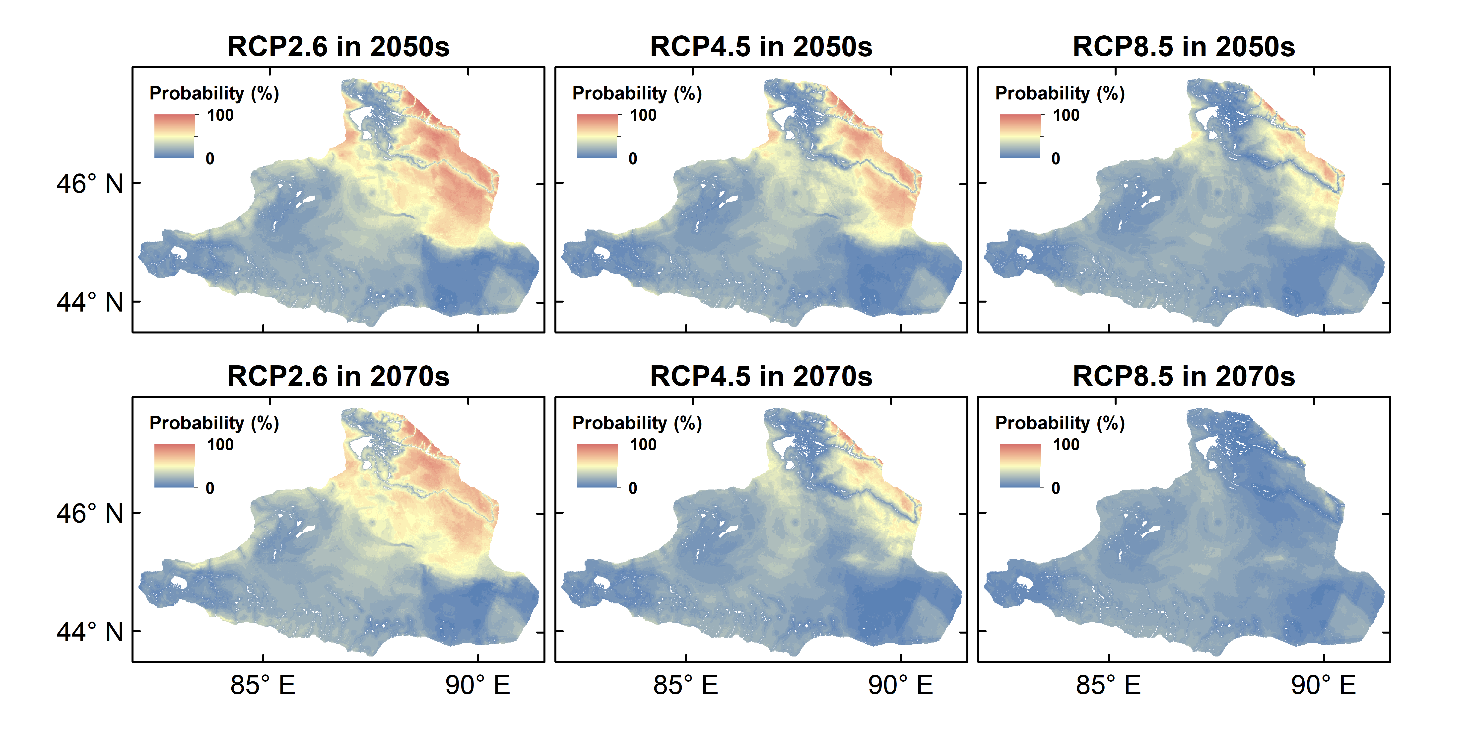
**Figure S4** Predicted future consensus maps of *Anabasis aphylla* in the Junggar Basin under three climate change scenarios (i.e. optimistic-RCP2.6; moderate-RCP4.5; pessimistic-RCP8.5) and two periods (i.e. 2050s and 2070s). Each map was generated from the predictions of all selected models based on all GCMs.


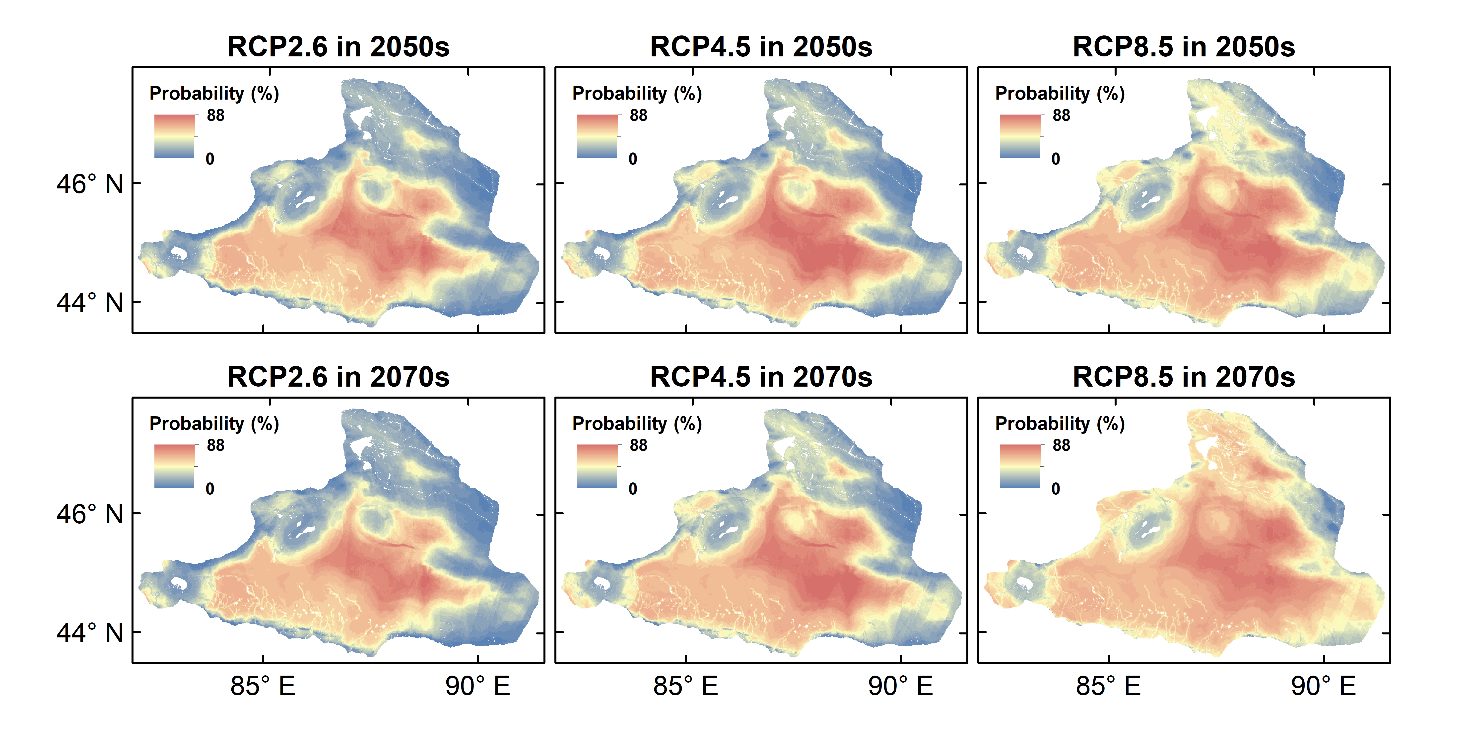
**Figure S5** Predicted future consensus maps of *Calligonum mongolicum* in the Junggar Basin under three climate change scenarios (i.e. optimistic-RCP2.6; moderate-RCP4.5; pessimistic-RCP8.5) and two periods (i.e. 2050s and 2070s). Each map was generated from the predictions of all selected models based on all GCMs.


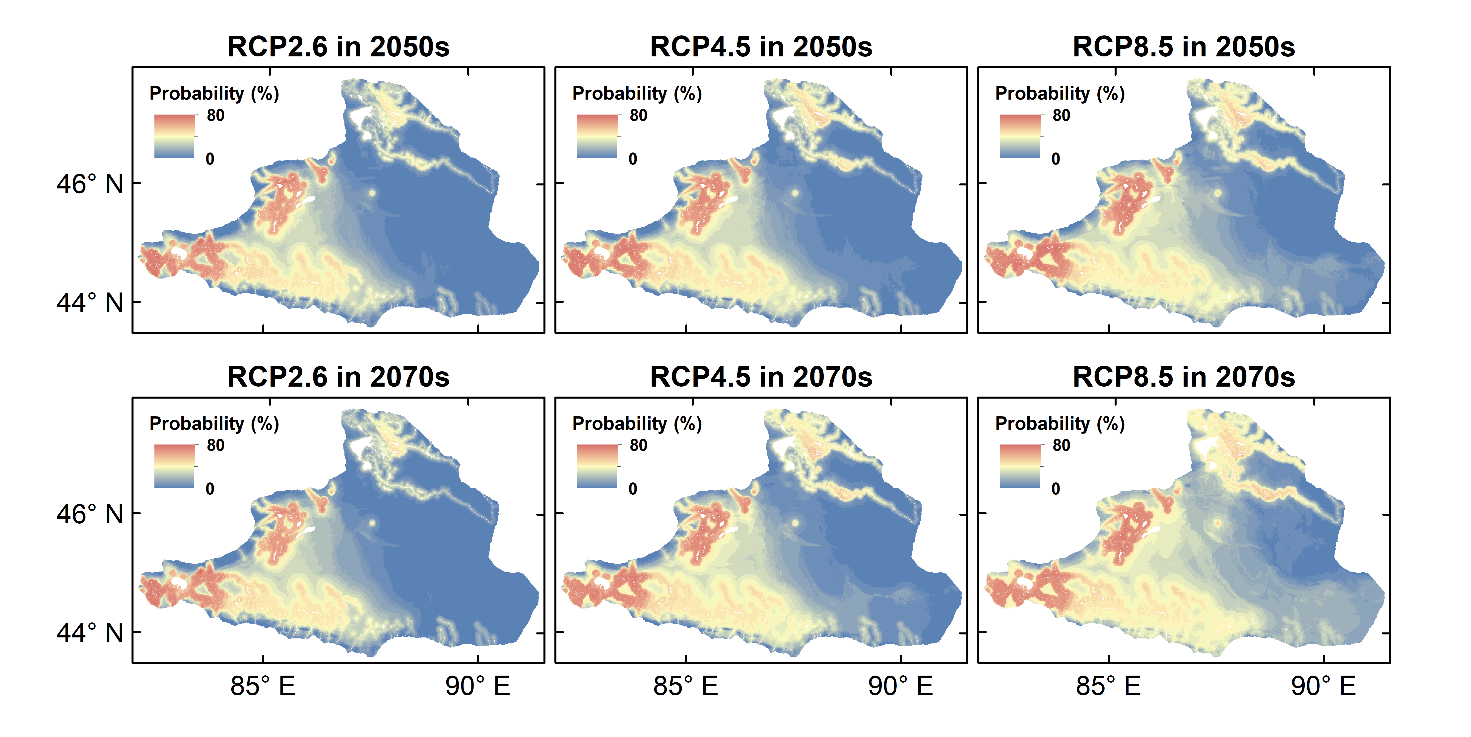
**Figure S6** Predicted future consensus maps of *Populus euphratica* in the Junggar Basin under three climate change scenarios (i.e. optimistic-RCP2.6; moderate-RCP4.5; pessimistic-RCP8.5) and two periods (i.e. 2050s and 2070s). Each map was generated from the predictions of all selected models based on all GCMs.

**Figure S7** The means of Mean Annual Temperature (MAT) change of the seven GCMs under three climate change scenarios (i.e. optimistic-RCP2.6; moderate-RCP4.5; pessimistic-RCP8.5) and two periods (i.e. 2050s and 2070s).


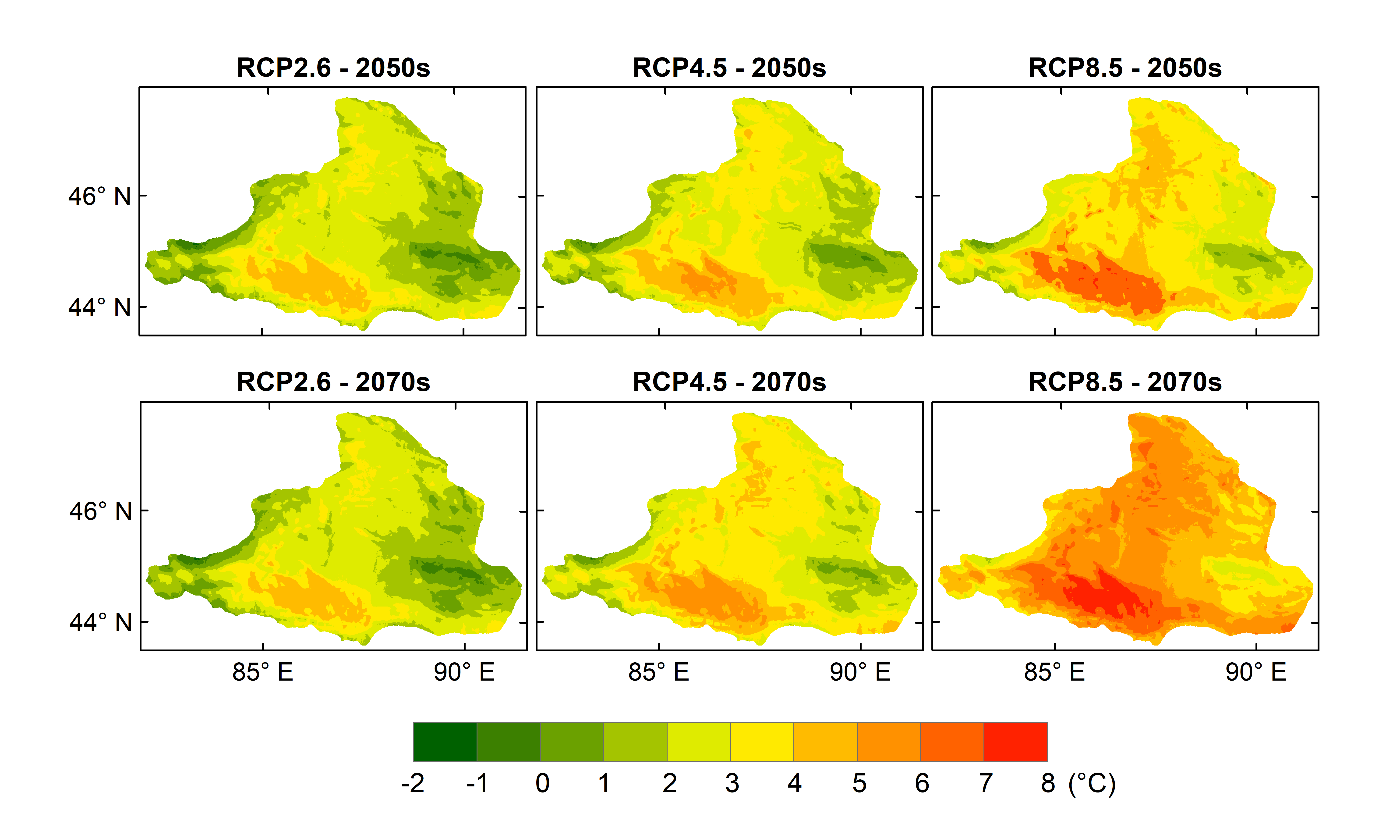


**Figure S8** The means of Mean Diurnal Range (MDR) change of the seven GCMs under three climate change scenarios (i.e. optimistic-RCP2.6; moderate-RCP4.5; pessimistic-RCP8.5) and two periods (i.e. 2050s and 2070s).


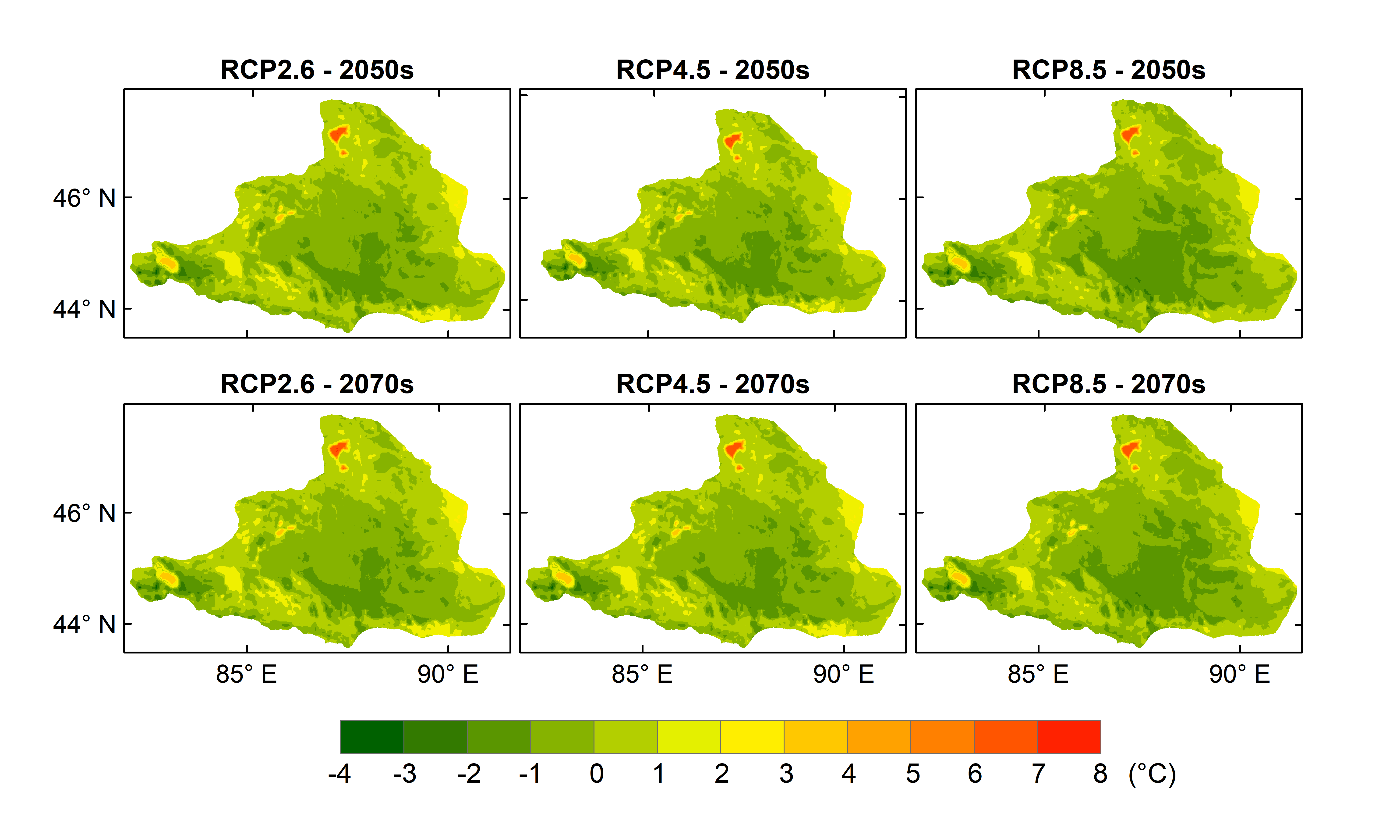


**Figure S9** The means of Isothermality (ISO) change of the seven GCMs under three climate change scenarios (i.e. optimistic-RCP2.6; moderate-RCP4.5; pessimistic-RCP8.5) and two periods (i.e. 2050s and 2070s).


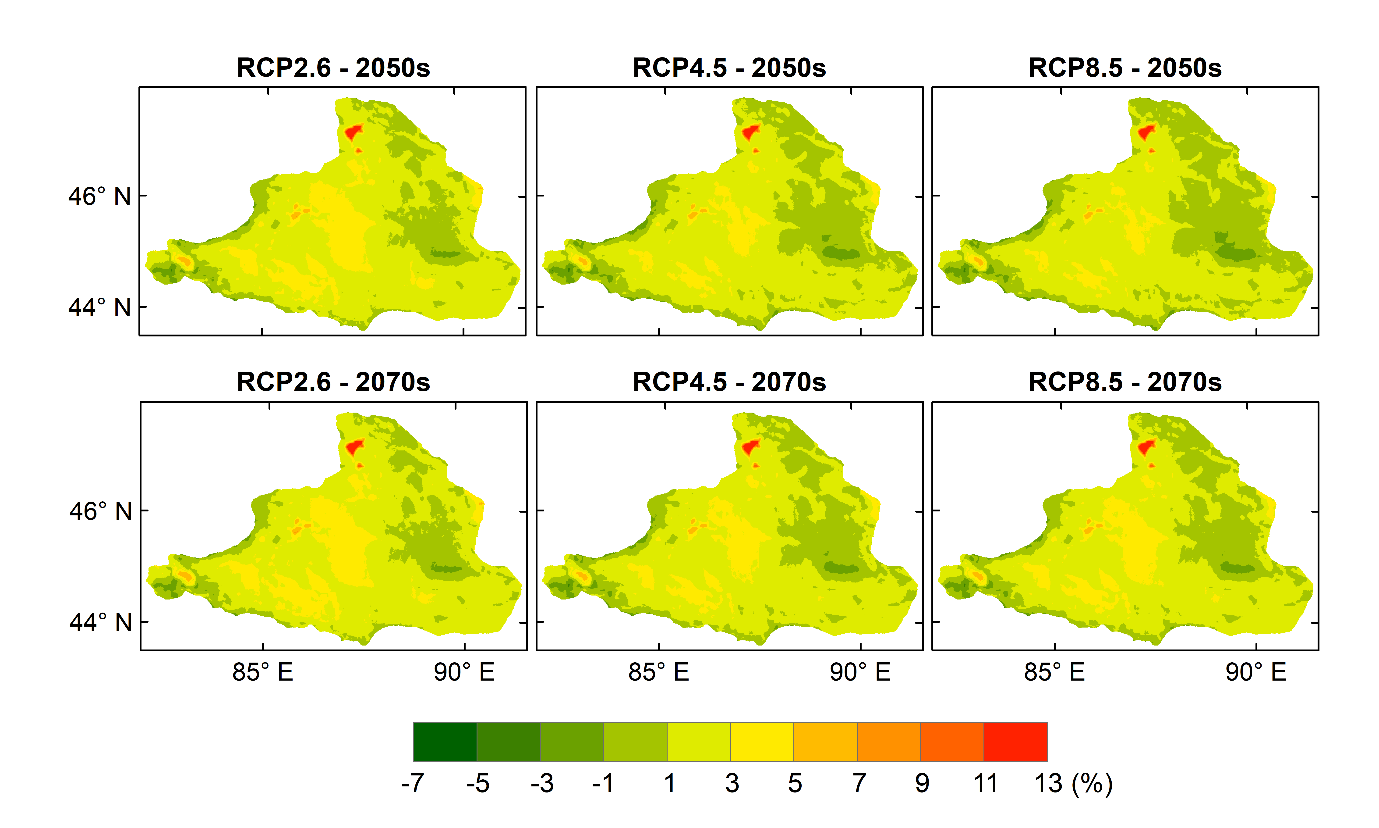


**Figure S10** The means of Min Temperature of Coldest Month (MTCM) change of the seven GCMs under three climate change scenarios (i.e. optimistic-RCP2.6; moderate-RCP4.5; pessimistic-RCP8.5) and two periods (i.e. 2050s and 2070s).


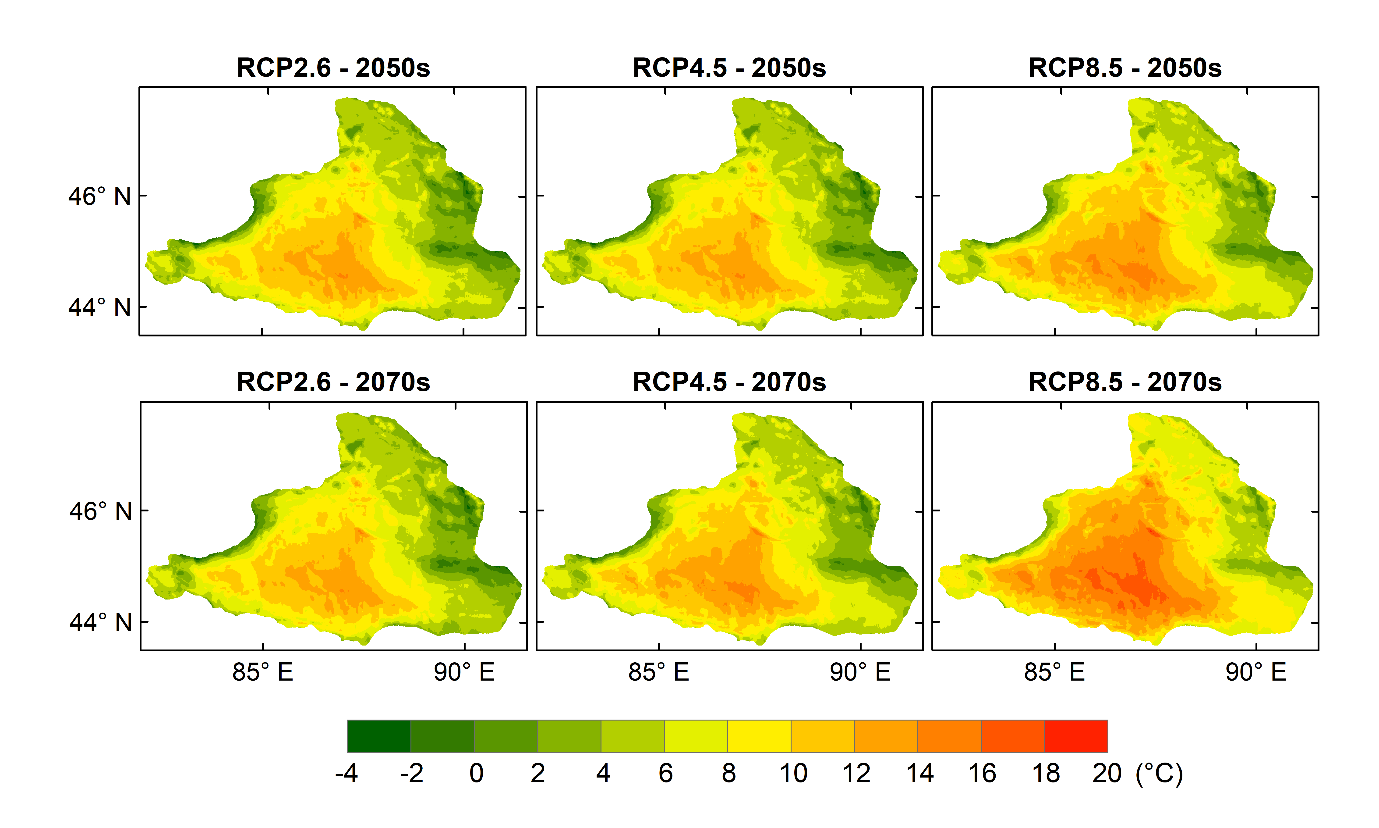


**Figure S11** The means of Precipitation of Wettest Month (PWM) change of the seven GCMs under three climate change scenarios (i.e. optimistic-RCP2.6; moderate-RCP4.5; pessimistic-RCP8.5) and two periods (i.e. 2050s and 2070s).


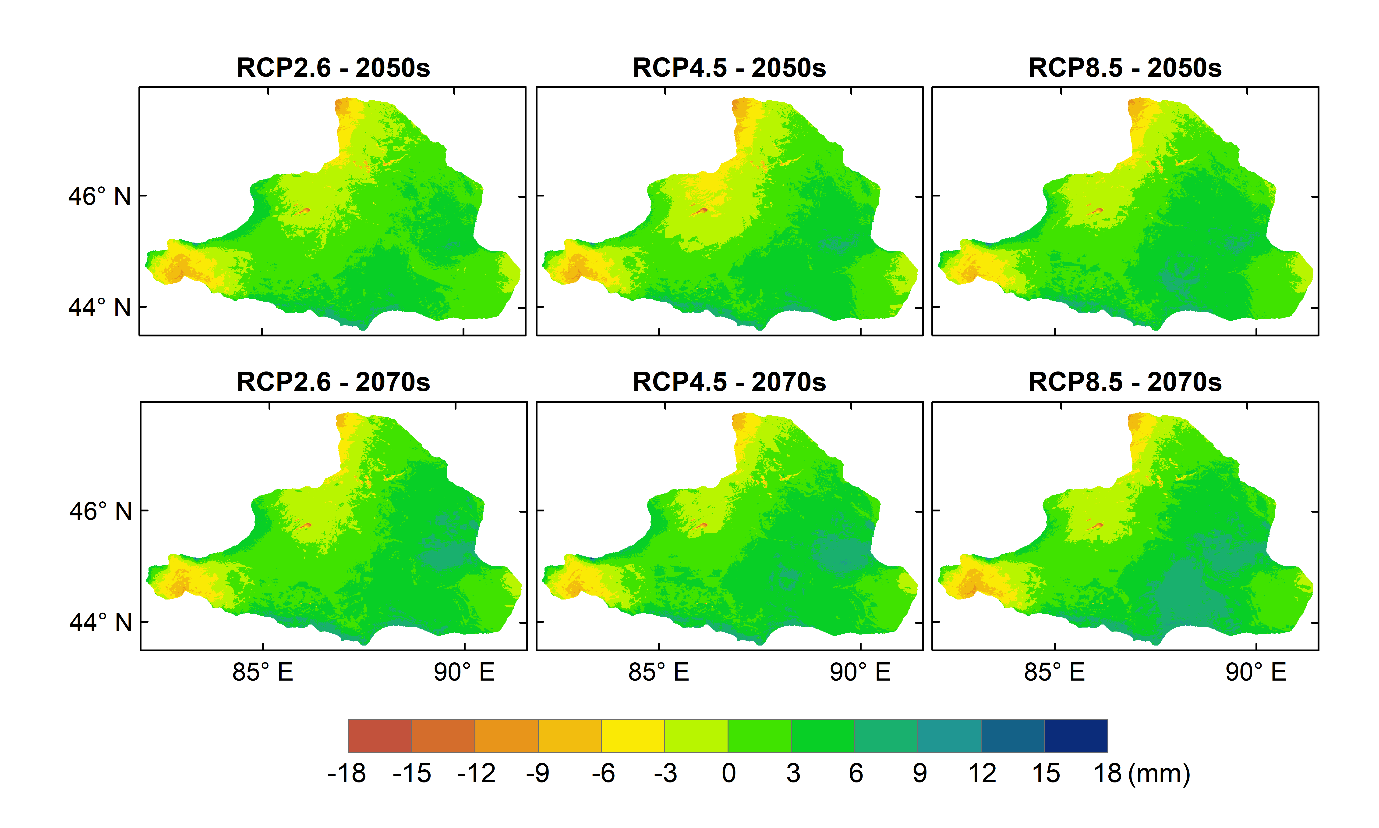


**Figure S12** The means of Precipitation of Coldest Quarter (PCQ) change of the seven GCMs under three climate change scenarios (i.e. optimistic-RCP2.6; moderate-RCP4.5; pessimistic-RCP8.5) and two periods (i.e. 2050s and 2070s).


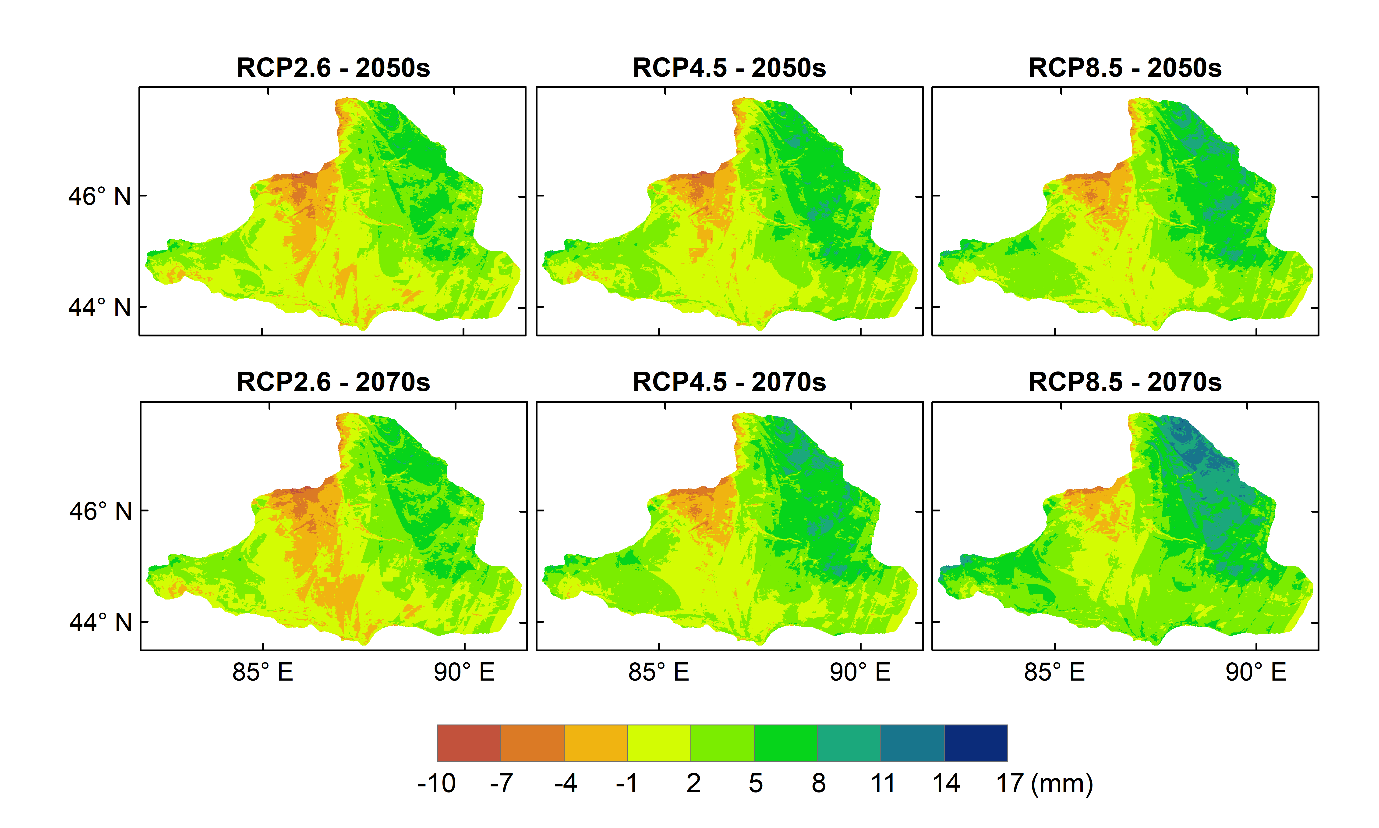

Supplement: Supplementary file 1 [file ECE3-9-13596-s001.docx]
